# Prevalence of dysphagia and risk of pneumonia and mortality in acute stroke patients: a meta-analysis

**DOI:** 10.1186/s12877-022-02960-5

**Published:** 2022-05-13

**Authors:** Kondwani Joseph Banda, Hsin Chu, Xiao Linda Kang, Doresses Liu, Li-Chung Pien, Hsiu-Ju Jen, Shu-Tai Shen Hsiao, Kuei-Ru Chou

**Affiliations:** 1 School of Nursing, College of Nursing, Taipei Medical University, Taipei, Taiwan; 2 Endoscopy Unit, Surgery Department, Kamuzu Central Hospital, Lilongwe, Malawi; 3 Institute of Aerospace and Undersea Medicine, School of Medicine, National Defense Medical Center, Taipei, Taiwan; 4 Department of Neurology, Tri-Service General Hospital, National Defense Medical Center, Taipei, Taiwan; 5 School of Nursing, University of Pennsylvania, Philadelphia, USA; 6 Department of Nursing, Wan Fang Hospital, Taipei Medical University, Taipei, Taiwan; 7 Center for Nursing and Healthcare Research in Clinical Practice Application, Wan Fang Hospital, Taipei Medical University, Taipei, Taiwan; 8 Post-Baccalaureate Program in Nursing, College of Nursing, Taipei Medical University, Taipei, Taiwan; 9 Psychiatric Research Center, Wan Fang Hospital, Taipei Medical University, Taipei, Taiwan; 10 Department of Nursing, Taipei Medical University-Shuang Ho Hospital, New Taipei, Taiwan; 11 Department of Nursing, Taipei Medical University Hospital, Taipei, Taiwan; 12 Psychiatric Research Center, Taipei Medical University Hospital, Taipei, Taiwan; 13 Neuroscience Research Center, Taipei Medical University, Taipei, Taiwan

**Keywords:** Acute Stroke, Dysphagia, Mortality, Pneumonia, Prevalence

## Abstract

**Background:**

Post-stroke dysphagia (PSD) has been associated with high risk of aspiration pneumonia and mortality. However, limited evidence on pooled prevalence of post-stroke dysphagia and influence of individual, disease and methodological factors reveals knowledge gap. Therefore, to extend previous evidence from systematic reviews, we performed the first meta-analysis to examine the pooled prevalence, risk of pneumonia and mortality and influence of prognostic factors for PSD in acute stroke.

**Methods:**

Our search was conducted in CINAHL, Cochrane Library, EMBASE, Ovid-Medline, PubMed, and Web of Science an initial search in October 2020 and a follow-up search in May 2021. Data synthesis was conducted using the Freeman-Tukey double-arcsine transformation model for the pooled prevalence rate and the DerSimonian-Lard random-effects model for prognostic factors and outcomes of PSD.

**Results:**

The pooled prevalence of PSD was 42% in 42 studies with 26,366 participants. PSD was associated with higher pooled odds ratio (OR) for risk of pneumonia 4.08 (95% CI, 2.13–7.79) and mortality 4.07 (95% CI, 2.17–7.63). Haemorrhagic stroke 1.52 (95% CI, 1.13–2.07), previous stroke 1.40 (95% CI, 1.18–1.67), severe stroke 1.38 (95% CI, 1.17–1.61), females 1.25 (95% CI, 1.09–1.43), and diabetes mellitus 1.24 (95% CI, 1.02–1.51) were associated with higher risk of PSD. Males 0.82 (95% CI, 0.70–0.95) and ischaemic stroke 0.54 (95% CI, 0.46–0.65) were associated with lower risk of PSD. Haemorrhagic stroke, use of instrumental assessment method, and high quality studies demonstrated to have higher prevalence of PSD in the moderator analysis.

**Conclusions:**

Assessment of PSD in acute stroke with standardized valid and reliable instruments should take into account stroke type, previous stroke, severe stroke, diabetes mellitus and gender to aid in prevention and management of pneumonia and thereby, reduce the mortality rate.

**Trial registration:**

https://osf.io/58bjk/?view_only=26c7c8df8b55418d9a414f6d6df68bdb.

**Supplementary information:**

The online version contains supplementary material available at 10.1186/s12877-022-02960-5.

## Background

Globally, 13.7 million strokes occur each year with 60% occurring in people under the age of 70 years [1]. Stroke is categorized into two major types including ischemic and hemorrhagic with three phases namely acute phase lasting from the initial onset of stroke until 2 weeks, sub-acute phase from greater than 2 weeks to 6 months, and chronic phase from greater than 6 months [2]. Current evidence from previous reviews reveals that the prevalence of post-stroke dysphagia (PSD) varies between 8.1% and 80% [3–7]. Higher rates of stroke have been observed in older age and females as such these factors may be linked with higher prevalence rate of PSD [6]. Furthermore, evidence shows that comorbidities including diabetes mellitus seem to play a role in the development of PSD in stroke [4, 6]. Moreover, disease characteristics including stroke type, location, and severity and previous stroke could also be associated with the occurence of PSD [7]. Various validated and non-validated assessment tools have been used for screening PSD in acute stroke with no consensus on the standard assessment tools [4]. Thus, the current study extends further knowledge from previous systematic reviews by conducting a meta-analysis using validated assessment tools and explore associated outcomes for acute stroke. In addition, the meta-analysis explores the influence of individual factors (age, gender and geographical location), disease factors (type, location, phase, severity of stroke, comorbidities and history of previous stroke), and methodological factors (assessment tools and study quality and design) on PSD in acute stroke.

Evidence on the pooled prevalence of PSD and influence of individual, disease and methodological factors in acute phase of stroke is lacking. Therefore, we conducted the first meta-analysis to determine the pooled prevalence, risk of pneumonia and mortality and influence of prognostic factors for PSD in acute stroke.

## Methods

### Search strategy and inclusion criteria

**Fig. 1:**
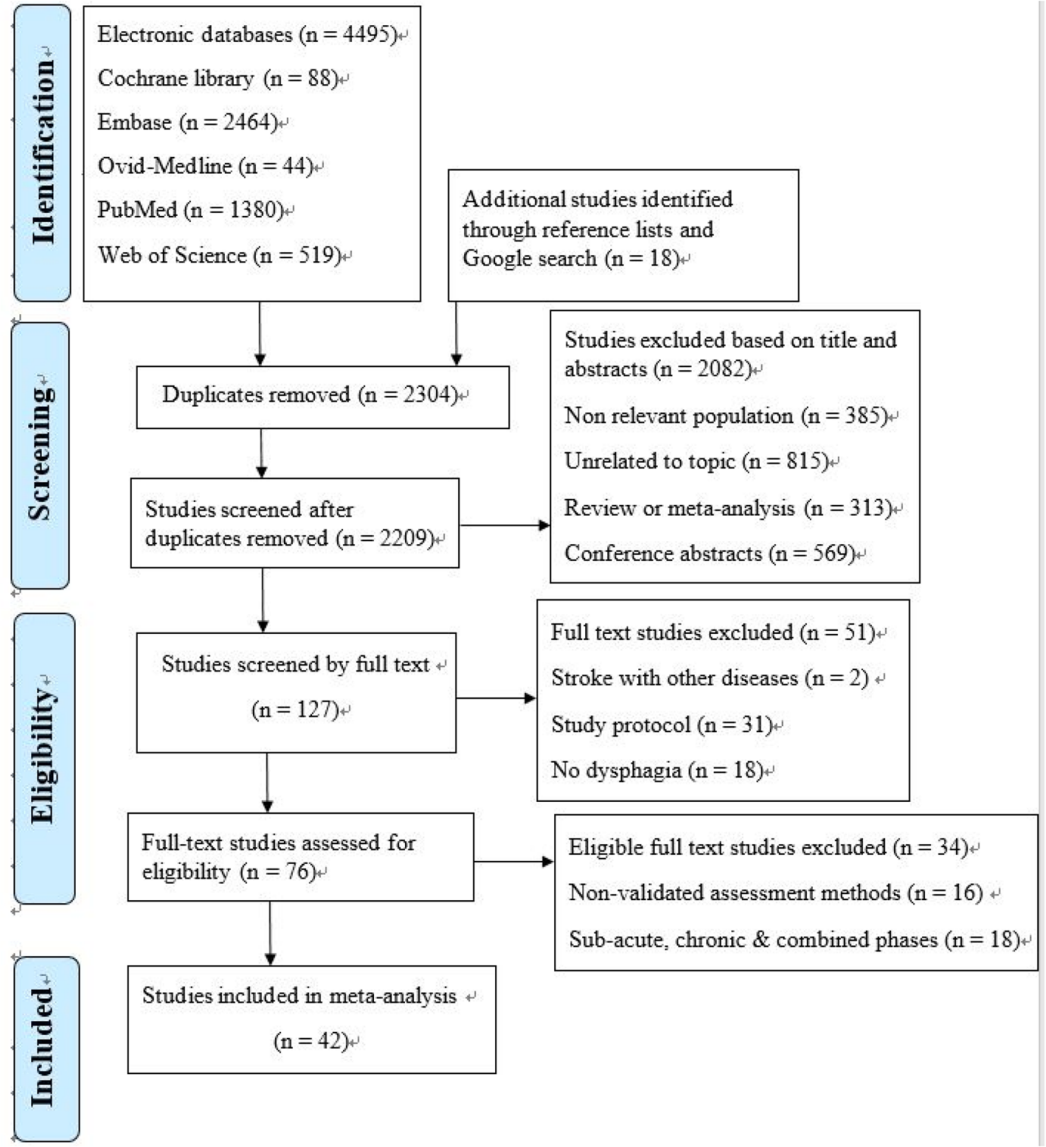
Flow chart for study selection

This meta-analysis study was conducted in accordance with the Meta-Analysis of Observational Studies in Epidemiology (MOOSE) guidelines [8, 9]. For scientific integrity, the study protocol was registered with Open Science Framework (OSF) with the link: https://osf.io/58bjk/?view_only=26c7c8df8b55418d9a414f6d6df68bdb. A comprehensive search was completed in PubMed, Web of Science, Embase, Cochrane Library, and Ovid-Medline from each database inception with an initial search in October 2020 and a follow-up search in May 2021. We used the following keywords in combination: prevalence of dysphagia, incidence of dysphagia, epidemiology of dysphagia, and stroke with a detailed search strategy in the supplementary material (Suppl. Table 1). We identified other potential studies by reviewing reference lists of previous systematic reviews and conducting a Google search (Fig. 1). Original authors were contacted when there was missing data in the published studies to include all eligible studies.

The included studies had to meet the following inclusion criteria without language restrictions (1) population: adults ≥ 18 years old with acute stroke (2) exposure of interest: dysphagia, (3) comparison: no dysphagia, (4) outcome of interest: epidemiology, incidence or prevalence, (5) study design: observational studies including prospective, retrospective and cross-sectional studies, (6) Studies used a validated diagnostic tool with sensitivity rates equal and greater than 90%.

### Ethics and consent to participate

The current meta-analysis used secondary data from participants in previously published studies in which consent was already sought from the participants. Thus, approval from IRB board and use of consent was not required for the current study.

### Data extraction and study outcomes

Two independent reviewers extracted data using standard data extraction forms with the following categories from the included studies focused on individual, disease, and methodological factors. The individual factors included author, year of publication, age, sample size, gender, country, and continent. The disease factors included type (haemorrhagic, ischaemic and combined), location (left and right hemisphere), severity and phase of stroke, comorbidities (diabetes mellitus and hypertension), atrial fibrillation, hyperlipidaemia, history of previous stroke, dysphagic and non-dysphagic acute stroke patients, stroke syndromes including LACS (lacunar syndrome), PACS (partial anterior circulation syndrome), POCS (posterior circulation syndrome), and TACS (total anterior circulation syndrome), and study outcomes including pneumonia and mortality. The methodological factors included assessment tool, time for baseline test and study design (Suppl. Table 2).

The primary outcome was pooled prevalence of PSD using validated instrumental assessment (1) Fiberoptic Endoscopic Evaluation of Swallowing (FEES) and (2) Videofluoroscopic Swallowing Study (VFSS), clinical objective assessment including (1) Volume-Viscosity Swallow Test (V-VST) and (2) Water Swallow Test (WST) and clinical subjective assessment including (1) Mann Assessment of Swallowing Ability (MASA) and (2) Standardized Swallowing Assessment (SSA). The secondary outcomes were (1) risk of pneumonia and (2) mortality. The prognostic factors of PSD were (1) gender (male and female), (2) stroke type (haemorrhagic and ischaemic), (3) stroke severity measured by National Institutes of Health Stroke Scale (NIHSS), (4) history of previous stroke, (5) comorbidities (hypertension and diabetes mellitus), (6) hyperlipidaemia, (7) atrial fibrillation, (8) stroke location (left and right hemisphere), and (8) stroke syndromes (LACS, PACS, POCS, and TACS).

### Quality assessment of included studies

The risk of bias was critically appraised using an assessment tool developed specifically for prevalence studies [10]. The external validity of the study domains is assessed in items 1–4 which includes selection and non-response bias. The internal validity of the study domains is assessed in items 5–10 which includes measurement bias (items 5–9), and bias related to the analysis (item 10). The overall risk of study bias is based on the summation of the nine items with the score for each item being 0 for low risk and 1 for high risk. The quality ranking is as follows 0–3: low risk study, 4–6: moderate risk study and 7–9: high risk study (Suppl. Table 3). Discrepancies among the reviewers were resolved through discussions with a third expert reviewer.

### Data synthesis and analysis

The Freeman-Tukey double-arcsine transformation model was used to estimate the pooled prevalence rates in R software with p_i_ as the proportion estimate from study *i* in the analysis (i = 1,…, N) [11, 12]. The pooled PSD prevalence estimate p_i_, was calculated as pi = e_i_/n_i_, with e_i_ being the number of PSD cases and n_i_ being the total sample size of the included studies, respectively. The Freeman-Tukey double-arcsine transformation model calculates weighted pooled estimates and then performs back-transformation on the pooled estimates to stabilize the within-study variance by using the binomial distribution. The Freeman–Tukey double-arcsine transformation equation:

y_i_ = g (p_i_) = arsin $\sqrt{ei/(ni+1)}$+ arsin $\sqrt{(ei+1)/(ni+1)}$ with variance v_i_ = 1/(n_i_ + 0.5). Publication bias was examined through visual inspection of the treatment estimates on the funnel plot [13]. The pooled estimates for PSD outcomes and association of prognostic factors were performed in Comprehensive Meta-Analysis (CMA), Version 2.0 software using the DerSimonian-Lard random-effects model with the inverse variance-weighted mean of the logarithm of OR with 95% confidence interval (CI) [14]. For the pooled OR of the outcomes and prognostic factors, the number of cases for each outcome or prognostic factor in acute stroke participants with dysphagia were compared to the number of cases for each outcome or prognostic factor in acute stroke participants without dysphagia.

### Heterogeneity assessment

Due to variations regarding individual, disease, and methodological factors among the included studies, statistical heterogeneity of prevalence estimates was examined using a ${\text{X}}^{2}-\text{b}\text{a}\text{s}\text{e}\text{d}$ test using Cochran’s Q statistic (*P* < 0.10) and the ${I}^{2}$statistic quantified heterogeneity indicating low, moderate and high heterogeneity with cut-off scores of 25%, 50%, and 75%, respectively [15].

### Moderator analysis

In order to identify possible modifying variables, we performed moderator analysis for the included studies [16]. Meta-regression was conducted for the continuous variable (mean age). Sub-group analysis was performed for categorical variables including (1) continent (Africa, Asia, Europe, North America, and South America), (2) stroke type (ischaemic, haemmorrhagic, and combined), (3) instrumental (VFSS/FEES), clinical objective (V-VST, and WST) and clinical subjective (MASA and SSA) assessment methods, (4) study quality (high and moderate), and (5) study design (prospective, cross-sectional, and retrospective).

## Results

### Study characteristics

We found 4,513 studies from the electronic databases, the Google search, and reference lists with 42 studies [13, 17–57] published between 1987 and 2021 being included in the analysis with one study reporting the assessment of PSD in haemorrhagic and ischaemic stroke separately (Fig. 1; Suppl. Table 2). We identified 26,336 acute stroke patients with 8,718 participants found to have dysphagia and the sample size ranged from 20 to 12,276. The mean age in the studies ranged from 50.4 to 78 (Suppl. Table 2).

### Prevalence of post-stroke dysphagia (PSD)

**Fig. 2:**
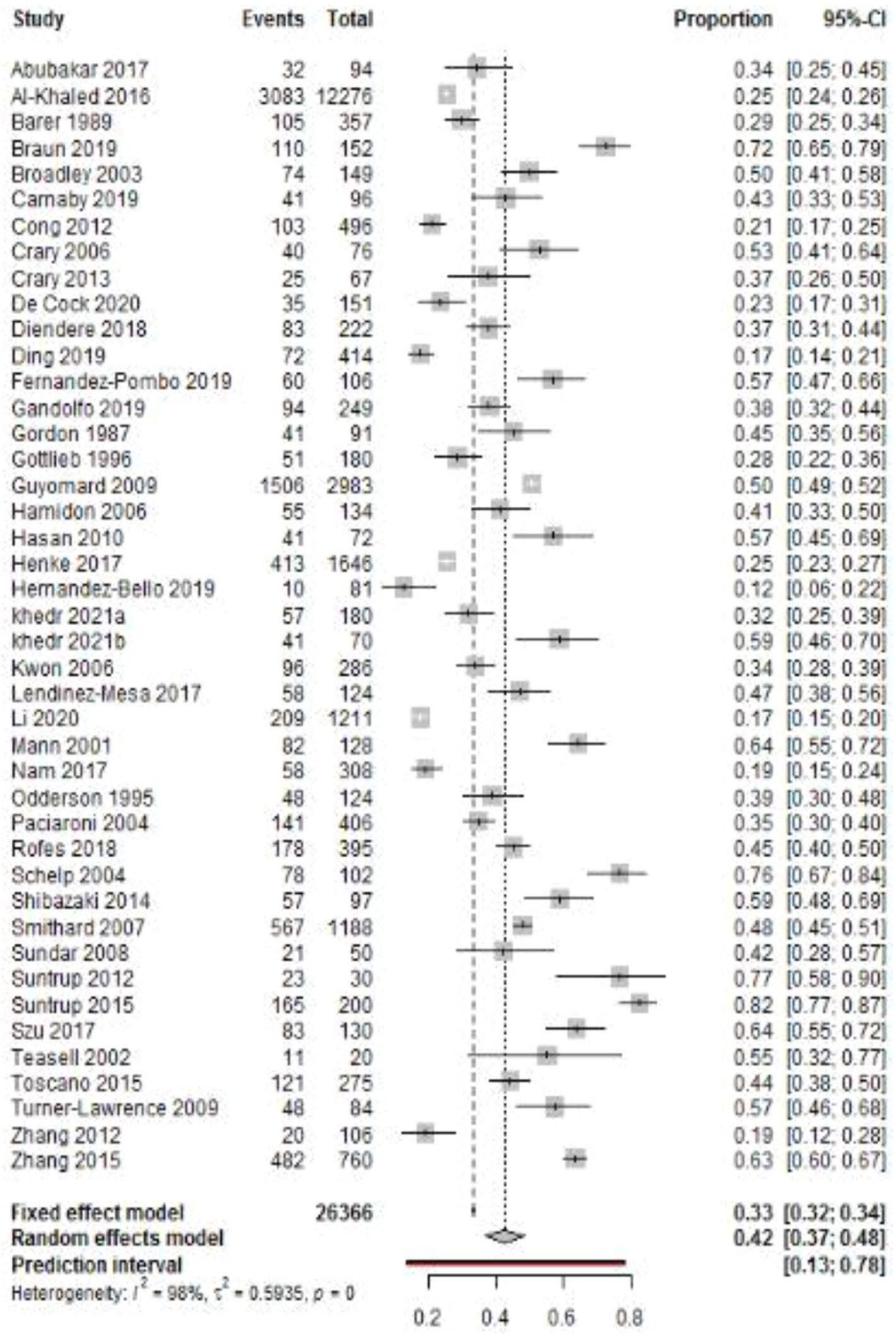
Pooled prevalence of post-stroke dysphagia

Our current study findings reveal the prevalence rate of PSD to be 42% (95%CI, 37–48%) with substantial heterogeneity (Fig. 2). The funnel plot revealed asymmetry of the study estimates and thus, showed evidence of publication bias (Suppl. Fig. 1). The prevalence rates by continents (*P* = 0.263) were as follows: South America: 61%, North America: 46%, Europe: 44%, Africa: 39%, and Asia: 37% (Suppl. Table 4).

### Risk of pneumonia and mortality with PSD

**Fig. 3:**
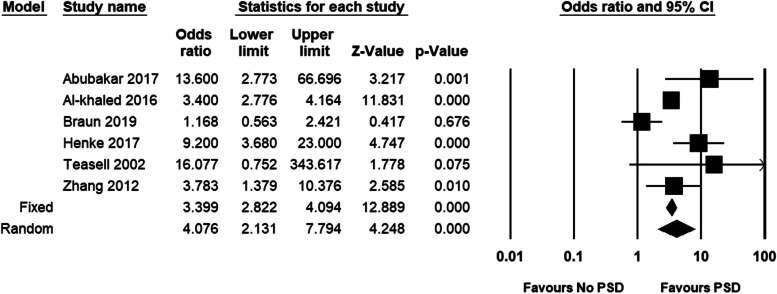
Post-stroke dysphagia and risk of pneumonia

**Fig. 4:**
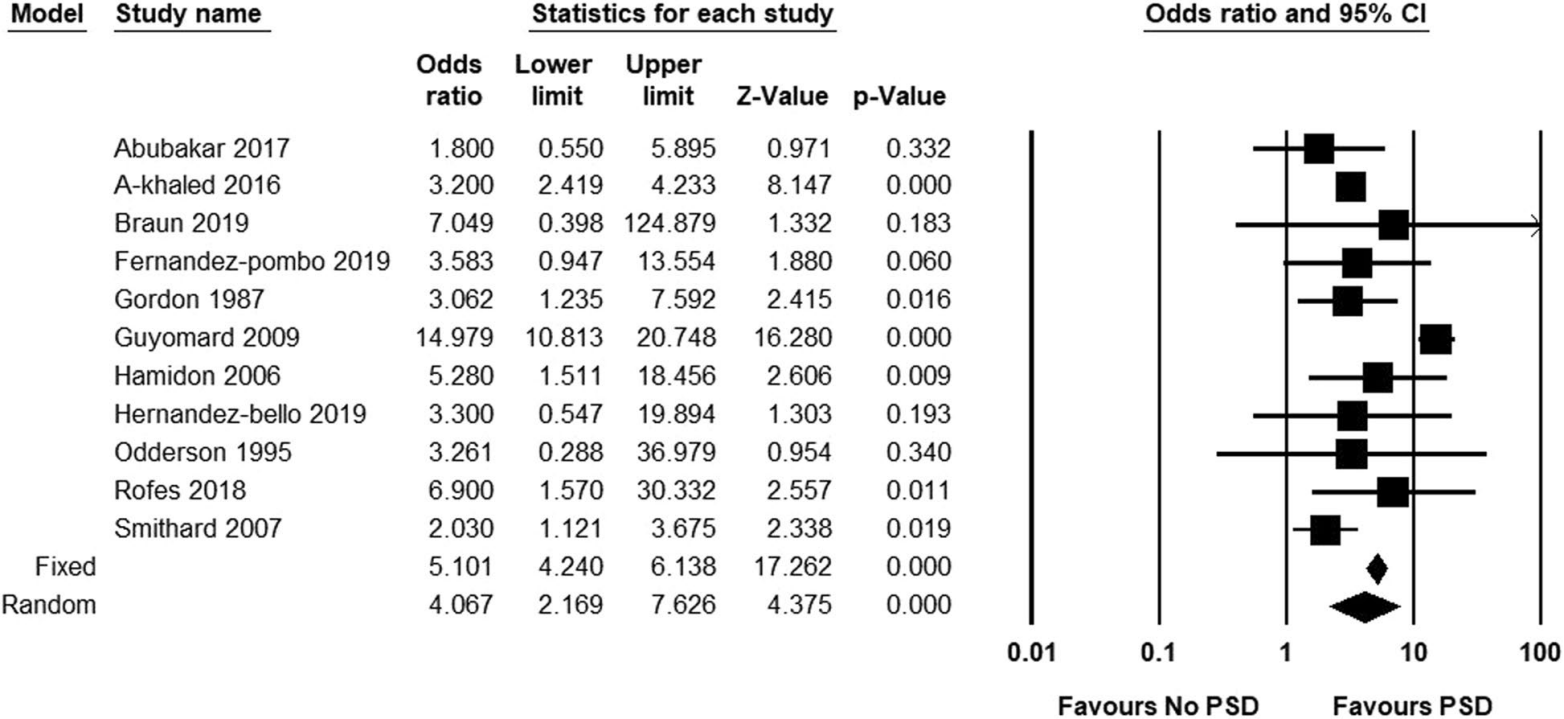
Post-stroke dysphagia and risk of mortality

The findings showed that the pooled odds ratio (OR) for risk of pneumonia was 4.08 (95% CI, 2.13–7.79). Participants with PSD were 4.35 times more likely at risk of pneumonia compared to participants without PSD (Fig. 3). Regarding mortality, the findings revealed that the pooled OR was 4.07 (95%CI, 2.17–7.63). Participants with PSD were 4.07 times more likely at risk of mortality compared to participants without PSD (Fig. 4).

### Prognostic factors for PSD

#### Gender

The study findings demonstrated that females were more likely to have PSD with a pooled OR of 1.25 (95%CI, 1.09–1.43) in 17 studies compared to males. Males with a pooled OR of 0.82 (95%CI, 0.70–0.95) were less likely at risk of PSD in 15 studies compared to females. The findings suggest that females have a higher risk of PSD compared males (Table 1).

**Table 1:** Prognostic factors for post-stroke dysphagia

Variables	n	OR (95% CI)	*p*-value
Gender			
Female	17	1.25 (1.09 – 1.43)	**0.002**
Male	15	0.82 (0.70–0.95)	**0.010**
Stroke type			
Haemorrhagic stroke	11	1.52 (1.13–2.04)	**0.006**
Ischaemic stroke	11	0.54 (0.46–0.65)	**0.000**
Previous stroke	6	1.40 (1.18–1.67)	**0.000**
Stroke severity	6	1.38 (1.17–1.61)	**0.000**
Comorbidities			
Diabetes Mellitus	10	1.24 (1.02–1.51)	**0.028**
Hypertension	10	1.16 (0.93–1.45)	0.179
Atrial fibrillation	5	1.66 (0.92–3.02)	0.094
Hyperlipidaemia	5	1.34 (0.92–1.95)	0.128
Stroke location			
Right hemisphere	7	1.14 (0.82–1.60)	0.434
Left hemisphere	7	0.93 (0.73–1.18)	0.521
Stroke syndromes			
TACS	6	1.81 (0.89–3.68)	0.104
PACS	5	1.06 (0.15–7.49)	0.951
POCS	5	0.70 (0.26–1.85)	0.472
LACS	5	0.67 (0.09–4.96)	0.696

*CI* confidence interval, *LACS *lacunar syndrome, *n* number of studies, *OR* odds ratio, *p*-value probability value, *PACS *partial anterior circulation syndrome, *POCS *posterior circulation syndrome, *TACS* total anterior circulation syndrome

### Previous stroke, stroke severity, type, location and syndromes, comorbidities, atrial fibrillation, and hyperlipidaemia in PSD

Regarding stroke type, the meta-analysis revealed that participants with haemorrhagic stroke had a pooled OR of 1.52 (95%CI, 1.13–2.07) compared to participants with ischaemic stroke. Participants with ischaemic stroke had a pooled OR of 0.54 (95%CI, 0.46–0.65) compared to participants with haemorrhagic stroke. Participants with haemorrhagic stroke were 1.52 times more likely at risk for PSD while participants with ischaemic stroke were 46% less likely at risk for PSD. Participants with history of previous stroke with a pooled OR of 1.40 (95%CI, 1.18–1.67) were 1.4 times more likely to have PSD compared to participants without previous history of stroke. Participants with a higher NIHSS score indicative of severe stroke with a pooled OR of 1.38 (95%CI, 1.17–1.61) were 1.38 times more likely to have PSD compared to participants with moderate and mild stroke. Acute stroke dysphagic participants with diabetes mellitus with a pooled OR of 1.24 (95%CI, 1.02–1.51) were 1.24 times more likely at risk of PSD compared to acute stroke non-dysphagic participants with diabetes mellitus.

However, hypertension, hyperlipidaemia, atrial fibrillation, right and left hemispheric stroke, and stroke syndromes revealed no significant difference in the risk of PSD in acute stroke patients. Acute stroke dysphagic participants with hypertension had a pooled OR of 1.16 (95%CI, 0.93–1.45) compared to acute stroke non-dysphagic participants with hypertension. Acute stroke dysphagic participants with hyperlipidaemia had a pooled OR of 1.34 (95%CI, 0.92–1.95) compared to acute stroke non-dysphagic participants with hyperlipidaemia. Acute stroke dysphagic participants with atrial fibrillation had a pooled OR of 1.66 (95%CI, 0.92–3.02) compared to acute stroke non-dysphagic participants with atrial fibrillation. Participants with right hemispheric stroke had a pooled OR of 1.14 (95%CI, 0.82–1.60) compared to participants with left hemispheric stroke. Participants with left hemispheric stroke had a pooled OR of 0.93 (95%CI, 0.73–1.18) compared to participants with right hemispheric stroke. LACS had a pooled OR of 0.67 (95%CI, 0.09–4.96), PACS had a pooled OR of 1.06 (95%CI, 0.15–7.49), POCS had a pooled OR of 0.70 (95%CI, 0.26–1.85), and TACS had a pooled OR of 1.81 (95%CI, 0.89–3.68) compared to other stroke syndromes in acute stroke patients (Table 1).

### Results of the moderator analysis

The findings of meta-regression demonstrated that age did not have any influence on the prevalence of PSD (*P =* 0.615). For every year increase in age, the study findings suggest a -1% non-significant decrease in the prevalence of PSD.

The participants’ primary diagnosis was stroke, with 15 studies reporting participants with ischaemic type, three studies reporting participants with haemorrhagic type, and 26 studies comprised of both ischaemic and haemorrhagic types. Regarding stroke type (*P* < 0.0001), studies which used participants with haemorrhagic stroke demonstrated to have a higher prevalence rate of 61% compared to studies with combined types of stroke with 49% and ischaemic stroke with 28% prevalence rates, respectively.

Regarding assessment method (*P* < 0.0001), instrumental assessment demonstrated to have a higher prevalence rate of 75% compared to clinical subjective assessment with 50% and clinical objective assessment with 38%  prevalence rates, respectively. Regarding study quality (*P* < 0.002), high quality studies showed to have a higher prevalence rate of 47 compared to moderate quality studies with 29% prevalence rate. Regarding the study design (*P* = 0.237), studies that used retrospective design revealed a lower prevalence rate with 32% compared to studies that used prospective design with 46% and cross-sectional design with 41% prevalence rates, respectively (Suppl. Table 4).

## Discussion

### Prevalence of PSD in acute stroke

To our knowledge, this is the first meta-analysis to provide a more precise estimation of prevalence of PSD in acute stroke patients at 42%. Previous evidence has shown that the prevalence of PSD ranged from 8.1 to 80% owing to use of validated and non-validated assessment tools [3–5]. The current findings revealed that stroke type and study quality in addition to assessment method contribute to the variation in the prevalence of PSD while age, continent and study design could not. Previous research has suggested that lesion size and location including internal capsule, primary sensory cortex, and insula of the right hemisphere and the brainstem play an important role in controlling the swallowing process [58]. Moreover, two of the included studies that assessed PSD in haemorrhagic stroke included participants with internal capsule and thalamic haemorrhage and this could explain the high prevalence [47, 50]. In addition, haemorrhagic stroke may lead to increased intracranial pressure as a result of increased area of bleeding in the acute phase of stroke causing disturbance in the cerebrospinal fluid circulation, which may further damage the swallowing neural network. However, the number of studies was limited and thus, more future studies are needed to better explain the association between haemorrhagic stroke and dysphagia. Instrumental assessment method revealed a high prevalence of PSD and is the recommended method for the diagnosis of PSD as it provides more objective assessment and results [7]. However, use of clinical objective and subjective assessment methods remains an important aspect of the clinical management of PSD complementing the instrumental assessment in clinical settings where the instrumental assessment is unavailable. Therefore, identification of reliable and standardized assessment methods for screening PSD in acute stroke patients to ensure reliable estimates should be considered. Furthermore, moderate quality studies demonstrated to underestimate the prevalence of PSD due to methodological flaws, which may lead to unreliable results. Thus, future high quality studies should be recommended for PSD assessment in acute stroke patients to provide reliable prevalence estimates.

### Risk of pneumonia and mortality with PSD

The current study revealed that acute stroke patients with PSD were 4.08 times likely at risk of developing pneumonia compared to acute stroke patients without PSD. Similarly, Eltringham et al., (2019) [59] using a systematic review found that stroke patients with dysphagia were at greater risk of developing stroke associated pneumonia compared to those without dysphagia [59]. Stroke has shown to cause paralysis and weakening of the pharynx, larynx and the soft palate causing further swallowing impairment in the pharyngeal phase [7]. As such, acute stroke patients with PSD are highly susceptible to silent aspiration and penetration, which further predisposes them to higher risk of aspiration pneumonia. Furthermore, stroke severity, which is one of the prognostic factors of PSD, has been associated with post-stroke pneumonia as it is linked to post-stroke immune impairment, which leads to immunosuppression mediated by the sympathetic nervous system in the brain [60]. This weakens the immune inflammatory response within the brain and possibly, the whole body putting acute stroke patients with severe stroke at higher risk of developing stroke-associated pneumonia. The risk of mortality was 4.07 times higher in acute stroke patients with PSD compared to acute stroke patients with no PSD. The possible explanation could be that acute stroke patients with PSD have a higher risk of developing pneumonia, which increases their risk of mortality. Therefore, assessment and management of PSD in acute stroke patients should take in account stroke severity to prevent development of pneumonia to improve their quality of life and consequently, reduce the mortality rates in this population.

### Prognostic factors for PSD

Significant gender differences were observed in the current meta-analysis as female participants demonstrated to have a higher risk compared to male participants with a lower risk of PSD. The study findings are inconsistent with previous studies, which found no significant gender differences in the risk of PSD [29, 35]. The possible explanation for this outcome would be that women have shown to have worse stroke-associated outcomes because they experience their first ever stroke at an older age compared to men [61]. Therefore, the findings that women are on higher risk for PSD compared to men may be explained by their age and a model, which diminishes the impact of age, should be used to explore the gender differences. The study findings showed that acute stroke patients with haemorrhagic stroke revealed to have a 1.52 times higher risk of PSD compared to acute stroke patients with ischaemic stroke who were 46% less likely at risk of PSD. The mechanism of higher risk of PSD has been largely attributed to stroke location and lesion size in the brain and haemmorrhagic strokes may have a higher impact and damage on the swallowing neural network compared to ischaemic stroke as a result of increased intracranial pressure, which causes disturbance in the cerebrospinal fluid flow in the brain [58]. However, haemmorrhagic strokes may differ in their sequelae along with volume of the intracerebral bleeding and the included studies in the current meta-analysis provided insufficient information regarding the subtypes and volume of the intracerebral hemorrhage. Thus, detailed investigation as regards to the subtypes, volume, and location of the haemmorrhagic or ischaemic stroke lesions in future studies might help further explain this association. Our findings also demonstrated that previous stroke was associated with 1.4 times risk of developing PSD in acute stroke patients. Previous evidence shows that previous stroke causes cumulative damage to the brain causing further impairment of the functional reserve and compensatory neural networks and therefore, PSD may be common in acute stroke patients with previous history of stroke [29]. Higher NIHSS score indicative of severe stroke represented a 1.38 times risk of PSD in acute stroke patients. The findings of the current meta-analysis are similar to previous study findings, which revealed that severe stroke is associated with high risk of PSD [59]. Patients with severe stroke may have severe impairment of the swallowing control centers in the brain, which affects the swallowing process leading to higher risk of developing of PSD. Furthermore, acute stroke patients with diabetes mellitus demonstrated to have a higher risk of PSD compared to those without diabetes mellitus [37]. Neuropathic factors linked to diabetes mellitus have been found to increase stroke morbidity, which may in turn lead to higher risk of PSD. However, biomedical risk factors for stroke including hypertension, hyperglycaemia (diabetes mellitus), and hyperlipidaemia may not be directly linked to development of PSD. Thus, factors likely to cause damage to the swallowing neural network following a stroke could help in explaining and understanding the link between dysphagia and stroke. Therefore, future prospective studies with more detailed demographic characteristics in acute stroke with and without dysphagia may help to clarify these associations.

### Strengths and limitations

Our study has several strengths. To our knowledge, this is the first meta-analysis to demonstrate the pooled prevalence of PSD in acute stroke patients and the effect of demographic factors using validated assessment tools. Secondly, to include all available studies without language limitations, we performed a comprehensive search. Thirdly, rigorous methodological procedures using MOOSE guidelines were followed and registered our study protocol with OSF. Lastly, we performed moderator analysis to identify possible sources of heterogeneity to help explain the variations among the included studies. However, despite these strengths, some limitations were observed in our study. First, heterogeneity was also observed in the study outcomes, however, a random-effects model and moderator analysis were conducted to account for the heterogeneity. Second, the associated outcomes and prognostic factors were based on authors judgement on the mostly reported factors in the included studies using unadjusted models, which may likely over-estimate or under-estimate the observed associations and, thus no causal relationship can be determined. Some studies could have missed owing to the presence of publication bias. Therefore, caution need to be taken in the interpretation of the results due to these limitations.

## Conclusions

In conclusion, this meta-analysis provides comprehensive evidence that the pooled prevalence of PSD is estimated at 42% associated with 4.08 times risk of pneumonia and a 4.07 times higher risk of mortality in acute stroke patients. Higher risk of PSD was associated with haemorrhagic stroke, previous stroke, severe stroke (higher NIHSS score), females, and diabetes mellitus while lower risk of PSD was observed in males and ischaemic stroke patients. Moderator analysis found that type of stroke, assessment method and study quality could explain the variation in the prevalence of PSD in acute stroke.

## Supplementary Information

**Additional file 1.**

## Data Availability

All related data materials have provided in the supplementary material and we have cited all eligible included studies.

## Abbreviations

CMA: Comprehensive Meta-Analysis
FEES: Fiberoptic Endoscopic Evaluation of Swallowing
LACS: Lacunar syndrome
MASA: Mann Assessment of Swallowing Ability
MOOSE: Meta-Analysis of Observational Studies in Epidemiology
NIHSS: National Institutes of Health Stroke Scale
OSF: Open Science Framework
PACS: Partial anterior circulation syndrome
POCS: Posterior circulation syndrome
SSA: Standardized Swallowing Assessment
TACS: Total anterior circulation syndrome
VFSS: Videofluoroscopic Swallowing Study
V-VST: Volume-Viscosity Swallow Test
WST: Water Swallow Test
