# Supplementary material for: Prevalence of dysphagia and risk of pneumonia and mortality in acute stroke patients: a meta-analysis
Source: BMC Geriatr. 2022 May 13;22:420. doi: 10.1186/s12877-022-02960-5 (PMC9103417; doi:10.1186/s12877-022-02960-5)
Supplement: Supplementary file 1 — Additional file 1. [file 12877_2022_2960_MOESM1_ESM.docx]

**Prevalence of dysphagia and risk of pneumonia and mortality in acute stroke patients: a meta-analysis**

**Authors:** Kondwani Joseph Banda, MSN, RNM^1,2^, Hsin Chu, MD, PhD^3,4^, Xiao Linda Kang, PhD, RN^1,5^, Doresses Liu, PhD, RN^1,6,7^, Li-Chung Pien, PhD, RN^8,11^, Hsiu-Ju Jen, MS, RN^1,9^, Shu-Tai H. Shen, MSN, RN^1,10^, Kuei-Ru Chou, PhD, RN, FAAN^1,7,9,11^

^1^ *School of Nursing, College of Nursing, Taipei Medical University, Taipei, Taiwan*

^2^ *Endoscopy Unit, Surgery Department, Kamuzu Central Hospital, Lilongwe, Malawi*

^3^ *Institute of Aerospace and Undersea Medicine, School of Medicine, National Defense Medical Center, Taipei, Taiwan*

^4^ *Department of Neurology, Tri-Service General Hospital, National Defense Medical Center, Taipei, Taiwan*

^5^ School of Nursing, University of Pennsylvania, USA

^6^ *Department of Nursing, Wan Fang Hospital, Taipei Medical University, Taipei, Taiwan*

^7^ *Center for Nursing and Healthcare Research in Clinical Practice Application, Wan Fang Hospital, Taipei Medical University, Taipei, Taiwan*

^8^ *Post-Baccalaureate Program in Nursing, College of Nursing, Taipei Medical University, Taipei, Taiwan*

^9^ *Department of Nursing, Taipei Medical University-Shuang Ho Hospital, New Taipei, Taiwan*

^10^ *Department of Nursing, Taipei Medical University Hospital, Taipei, Taiwan*

^11^ *Psychiatric Research Center, Taipei Medical University Hospital, Taipei, Taiwan*

Supplementary material

**Supplemental Table 1:** Search strategy

**Supplemental Table 2:** Study characteristics

**Supplemental Table 3:** Risk of bias assessment

**Supplemental Table 4:** Moderator analysis

**Supplemental Figure 1:** Funnel plot for publication bias

**Supplemental Figure 2:** Gender – female

**Supplemental Figure 3:** Gender – male

**Supplemental Figure 4:** Haemorrhagic stroke

**Supplemental Figure 5:** Ischaemic stroke

**Supplemental Figure 6:** Previous stroke

**Supplemental Figure 7:** Stroke severity

**Supplemental Figure 8:** Diabetes mellitus

**Supplemental Figure 9:** Hypertension

**Supplemental Figure 10:** Atrial fibrillation

**Supplemental Figure 11:** Hyperlipidaemia

**Supplemental Figure 12:** Right hemisphere

**Supplemental Figure 13:** Left hemisphere

**Supplemental Figure 14:** Total anterior circulation syndrome (TACS)

**Supplemental Figure 15:** Partial anterior circulation syndrome PACS

**Supplemental Figure 16:** Posterior circulation syndrome (POCS)

**Supplemental Figure 17:** Lacunar syndrome (LACS)

**Supplemental References**

**Supplemental Table 1:** Search strategy

| Database | Keywords | Number of studies |
| --- | --- | --- |
| Cochrane Library | prevalence of dysphagia in Title Abstract Keyword AND stroke in Title Abstract Keyword - (Word variations have been searched) (10 studies)  incidence of dysphagia in Title Abstract Keyword AND stroke in Title Abstract Keyword - (Word variations have been searched) (48 studies)  epidemiology of dysphagia in Title Abstract Keyword AND stroke in Title Abstract Keyword - (Word variations have been searched) (30 studies) | 88 |
| Embase | prevalence of dysphagia AND stroke (401 studies)  incidence of dysphagia AND stroke (542 studies)  epidemiology of dysphagia AND stroke (1521 studies) | 2464 |
| Medline | prevalence of dysphagia AND stroke.mp. [mp=title, abstract, original title, name of substance word, subject heading word, floating sub-heading word, keyword heading word, organism supplementary concept word, protocol supplementary concept word, rare disease supplementary concept word, unique identifier, synonyms] (23 studies)  incidence of dysphagia AND stroke. mp. [mp=title, abstract, original title, name of substance word, subject heading word, floating sub-heading word, keyword heading word, organism supplementary concept word, protocol supplementary concept word, rare disease supplementary concept word, unique identifier, synonyms] (19 studies)  epidemiology of dysphagia AND stroke. mp. [mp=title, abstract, original title, name of substance word, subject heading word, floating sub-heading word, keyword heading word, organism supplementary concept word, protocol supplementary concept word, rare disease supplementary concept word, unique identifier, synonyms] (2 studies) | 44 |
| PubMed | prevalence of dysphagia AND stroke (483 studies)  incidence of dysphagia AND stroke (503 studies)  epidemiology of dysphagia AND stroke (394 studies) | 1380 |
| Web of Science | prevalence of dysphagia AND stroke Timespan: All years. Indexes: SCI-EXPANDED, SSCI. (267 studies)  incidence of dysphagia AND stroke Timespan: All years. Indexes: SCI-EXPANDED, SSCI. (216 studies)  epidemiology of dysphagia AND stroke Timespan: All years. Indexes: SCI-EXPANDED, SSCI. (36 studies) | 519 |

**Supplemental Table 2:** Study characteristics

| Author (Year) | Age  Sample size | Type and Phase of Stroke | Design  Country & Continent | Diagnostic Method | Prevalence | Prognostic Factors | Outcomes |
| --- | --- | --- | --- | --- | --- | --- | --- |
| Abubakar & Jamoh, 2017  [1] | Age  55.5 (15.7)  Sample size  Total: 94  Male: 53  Female: 41 | Type  Ischaemic  Haemorrhagic  Phase  Acute | Design  Prospective study  Nigeria, Africa | WST  Baseline Test  < 72 hours | Dysphagia  (n = 32)  No dysphagia  (n = 62) | Diabetes mellitus  Hypertension | Pneumonia  Mortality |
| Al-Khaled et al., 2016 [2] | Age  73 (13)  Sample size  Total: 12276  Male: 6261  Female: 6015 | Type  Ischaemic  Phase  Acute | Design  Prospective study  Germany, Europe | WST  Baseline Test  < 3 hours | Dysphagia  (n = 3083)  No dysphagia  (n = 9193) | Previous stroke  Diabetes mellitus  Hypertension | Pneumonia  Mortality |
| Barer 1989 [3] | Age  70  Sample size  Total: 357  Male: 189  Female: 168 | Type  Ischemic  Hemorrhagic  Phase  Acute | Design  Prospective study  Britain, Europe | WST  Baseline Test  < 48 hours | Dysphagia  (n = 105)  No dysphagia  (n = 252) | NA | NA |
| Braun et al., 2019 [4] | Age  73 (61.3-81)  Sample size  Total: 152  Male: 94  Female: 58 | Type  Ischaemic  Haemorrhagic  Phase  Acute | Design  Prospective study  Germany, Europe | FEES  Baseline Test  NA | Dysphagia  (n = 110)  No dysphagia  (n = 42) | NA | Pneumonia  Mortality |
| Broadley et al., 2003 [5] | Age  72  Sample size  Total: 149  Male: 88  Female: 61 | Type  Ischaemic  Phase  Acute | Design  Prospective study  Australia, Oceania | WST  Baseline Test  < 72 hours | Dysphagia  (n = 74)  No dysphagia  (n = 75) | NA | NA |
| Carnaby, Sia & Crary, 2019  [6] | Age  62.5 (14.1)  Sample size  Total: 96  Male: 58  Female: 38 | Type  Ischaemic  Haemorrhagic  Phase  Acute | Design  Prospective study  USA, North America | MASA  Baseline Test  < 24 hours | Dysphagia  (n = 41)  No dysphagia  (n = 55) | Stroke severity | NA |
| Cong et al.,2012 [7] | Age  65 (12)  Sample size  Total: 496  Male: 251  Female: 245 | Type  Ischaemic  Phase  Acute | Design  Retrospective study  China, Asia | WST  Baseline Test  < 72 hours | Dysphagia  (n = 103)  No dysphagia  (n = 393) | Diabetes mellitus  Hypertension | NA |
| Crary et al., 2006 [8] | Age  66.2 (11.8)  Sample size  Total: 76  Male: 36  Female: 40 | Type  Ischaemic  Phase  Acute | Design  Prospective study  USA, North America | MASA  Baseline Test  < 72 hours | Dysphagia  (n = 40)  No dysphagia  (n = 36) | Stroke severity  Previous stroke  Hyperlipidaemia  LACS  POCS  TACS | NA |
| Crary et al., 2013 [9] | Age  65.7  Sample size  Total: 67  Male: 29  Female: 38 | Type  Ischaemic    Phase  Acute | Design  Prospective study  USA, North America | MASA  Baseline Test  < 48 hours | Dysphagia  (n = 25)  No dysphagia  (n = 42) | Stroke severity  Diabetes mellitus  Hypertension  Hyperlipidaemia  LACS  PACS  POCS  TACS | NA |
| De Cock et al., 2020 [10] | Age  67 (14)  Sample size  Total: 151  Male: 84  Female: 67 | Type  Ischaemic  Haemorrhagic  Phase  Acute | Design  Prospective study  Belgium, Europe | WST  Baseline Test  < 72 hours | Dysphagia  (n = 35)  No dysphagia  (n = 116) | Stroke severity  Left hemisphere  Right hemisphere | NA |
| Diendere et al., 2018 [11] | Age  60.5 (14.2)  Sample size  Total: 222  Male: 121  Female: 101 | Type  Ischaemic  Haemorrhagic  Phase  Acute | Design  Prospective study  Burkina Faso, Africa | WST  Baseline Test  < 24 hours | Dysphagia  (n = 83)  No dysphagia  (n = 139) | NA | NA |
| Ding et al., 2019 [12] | Age  71.5 (50-89)  Sample size  Total: 414  Male: 264  Female: 150 | Type  Ischaemic  Haemorrhagic  Phase  Acute | Design  Retrospective study  China, Asia | WST  Baseline Test  < 48 hours | Dysphagia  (n = 72)  No dysphagia  (n = 342) | NA | NA |
| Fernandez-Pombo et al., 2019 [13] | Age  72.1 (13.5)  Sample size  Total: 106  Male: 58  Female: 48 | Type  Ischaemic  Haemorrhagic  Phase  Acute | Design  Prospective study  Spain, Europe | V-VST  Baseline Test  < 72 hours | Dysphagia  (n = 60)  No dysphagia  (n = 46) | Left hemisphere  Right hemisphere | Mortality |
| Gandolfo et al., 2019 [14] | Age  74  Sample size  Total: 249  Male: 126  Female: 123 | Type  Ischaemic  Haemorrhagic  Phase  Acute | Design  Prospective study  Italy, Europe | WST  Baseline Test  < 7 days | Dysphagia  (n = 94)  No dysphagia  (n = 155) | NA | NA |
| Gordon, Hewer & Wade, 1987  [15] | Age  70  Sample size  Total: 91  Male: 38  Female: 53 | Type  Ischaemic  Haemorrhagic  Phase  Acute | Design  Prospective study  Italy, Europe | WST  Baseline Test  < 48 hours | Dysphagia  (n = 41)  No dysphagia  (n = 50) | Previous stroke  Diabetes mellitus  Hypertension | Mortality |
| Gottlieb et al., 1996 [16] | Age  74 (8)  Sample size  Total: 180  Male: 86  Female: 94 | Type  Ischaemic  Haemorrhagic  Phase  Acute | Design  Prospective study  Israel, Middle East | WST  Baseline Test  < 48 hours | Dysphagia  (n = 51)  No dysphagia  (n = 129) | NA | NA |
| Guyomard et al., 2009 [17] | Age  78 (17-105)  Sample size  Total: 2983  Male: 1333  Female: 1650 | Type  Ischaemic  Haemorrhagic  Phase  Acute | Design  Retrospective study  United Kingdom, Europe | WST  Baseline Test  < 48 hours | Dysphagia  (n = 1506)  No dysphagia  (n = 1477) | Previous stroke  LACS  PACS  POCS  TACS | Mortality |
| Hamidon, Nabil & Raymond, 2006 [18] | Age  64.4 (10.9)  Sample size  Total: 134  Male: 67  Female: 67 | Type  Ischaemic  Phase  Acute | Design  Prospective study  Malaysia, Asia | WST  Baseline Test  < 7 days | Dysphagia  (n = 55)  No dysphagia  (n = 79) | Diabetes mellitus  Hypertension | Mortality |
| Hasan et al., 2010 [19] | Age  60.6 (30-82)  Sample size  Total: 72  Male: 40  Female: 32 | Type  Ischaemic  Haemorrhagic  Phase  Acute | Design  Prospective study  Iraq, Asia | MASA  Baseline Test  < 7 days | Dysphagia  (n = 41)  No dysphagia  (n = 31) | Left hemisphere  Right hemisphere  PACS  TACS | NA |
| Henke, Foerch & Lapa, 2017  [20] | Age  70 (14)  Sample size  Total: 1646  Male: 914  Female: 732 | Type  Ischaemic  Phase  Acute | Design  Prospective cohort study  Germany, Europe | WST  Baseline Test  < 7 days | Dysphagia  (n = 413)  No dysphagia  (n = 1029) | NA | Pneumonia |
| Hernandez-Bello, Castellot-Perales & Tomas-Aznar, 2018 [21] | Age  72.8 (12.5)  Sample size  Total: 81  Male: 53  Female: 28 | Type  Ischemic  Haemorrhagic    Phase  Acute | Design  Prospective study  Spain, Europe | V-VST  Baseline Test  < 24 hours | Dysphagia  (n = 10)  No dysphagia  (n = 71) | Previous stroke | Mortality |
| Khedr et al., 2021a [22] | Age  62.8 (12.1)  Sample size  Total: 180  Male: 84  Female: 96 | Type  Ischaemic    Phase  Acute | Design  Cross-sectional study  Egypt, Africa | WST  Baseline Test  < 72 hours | Dysphagia  (n = 57)  No dysphagia  (n = 123) | Diabetes mellitus  Hypertension  Hyperlipidaemia  Left hemisphere  Right hemisphere | NA |
| Khedr et al., 2021b [22] | Age  50.4 (13.3)  Sample size  Total: 70  Male: 32  Female: 38 | Type  Haemorrhagic    Phase  Acute | Design  Cross-sectional study  Egypt, Africa | WST  Baseline Test  < 72 hours | Dysphagia  (n = 41)  No dysphagia  (n = 70) | Diabetes mellitus  Hypertension  Hyperlipidaemia  Left hemisphere  Right hemisphere | NA |
| Kwon et al., 2006 [23] | Age  62.8 (12.1)  Sample size  Total: 286  Male: 192  Female: 94 | Type  Ischaemic  Haemorrhagic  Phase  Acute | Design  Prospective study  Korea, Asia | WST  Baseline Test  < 5 days | Dysphagia  (n = 96)  No dysphagia  (n = 190) | NA | NA |
| Lendinez-Mesa et al., 2016 [24] | Age  56.5 (12.4)  Sample size  Total: 124  Male: 88  Female: 36 | Type  Ischaemic  Haemorrhagic  Phase  Acute | Design  Cross-sectional study  Spain, Europe | V-VST  Baseline Test  < 24 hours | Dysphagia  (n = 58)  No dysphagia  (n = 66) | NA | NA |
| Li et al., 2019  [25] | Age  63.5 (13.7)  Sample size  Total: 1211  Male: 801  Female: 410 | Type  Ischaemic  Phase  Acute | Design  Retrospective study  China, Asia | WST  Baseline Test  < 24 hours | Dysphagia  (n = 209)  No dysphagia  (n = 1002) | NA | NA |
| Mann, Hankey & Cameron, 2001 [26] | Age  71 (12.1)  Sample size  Total: 128  Male: 81  Female: 47 | Type  Ischemic  Haemorrhagic  Phase  Acute | Design  Prospective study  Australia, Oceania | WST  Baseline Test  < 72 hours | Dysphagia  (n = 82)  No dysphagia  (n = 46) | Left hemisphere  Right hemisphere  LACS  PACS  POCS  TACS | NA |
| Nam et al., 2017 [27] | Age  68  Sample size  Total: 308  Male: 192  Female: 66 | Type  Ischaemic    Phase  Acute | Design  Retrospective study  South Korea, Asia | WST  Baseline Test  < 48 hours | Dysphagia  (n = 58)  No dysphagia  (n = 250) | NA | NA |
| Odderson et al., 1995 [28] | Age  75.3  Sample size  Total: 124  Male: 49  Female: 75 | Type  Ischaemic  Phase  Acute | Design  Prospective study  USA, North America | WST  Baseline Test  < 24 hours | Dysphagia  (n = 48)  No dysphagia  (n = 76) | NA | Mortality |
| Paciaroni et al., 2004 [29] | Age  73.2 (11.4)  Sample size  Total: 406  Male: 222  Female: 184 | Type  Ischaemic  Haemorrhagic  Phase  Acute | Design  Prospective study  Italy, Europe | WST  Baseline Test  < 24 hours | Dysphagia  (n = 141)  No dysphagia  (n = 265) | Diabetes mellitus  Hypertension  Hyperlipidaemia  Left hemisphere  Right hemisphere | NA |
| Rofes et al., 2017 [30] | Age  73.2 (13.1)  Sample size  Total: 395  Male: 211  Female: 184 | Type  Ischaemic  Haemorrhagic  Phase  Acute | Design  Prospective study  Brazil, South America | V-VST  Baseline Test  < 24 hours | Dysphagia  (n = 178)  No dysphagia  (n = 217) | Stroke severity  Previous stroke  Diabetes mellitus  Hypertension  Hyperlipidaemia  Left hemisphere  Right hemisphere | Mortality |
| Schelp et al., 2004 [31] | Age  62.2 (32-92)  Sample size  Total: 102  Male: 66  Female: 36 | Type  Ischaemic  Haemorrhagic  Phase  Acute | Design  Prospective study  Brazil, South America | WST  Baseline Test  < 72 hours | Dysphagia  (n = 78)  No dysphagia  (n = 24) | NA | NA |
| Shibazaki et al., 2014 [32] | Age  68.1  Sample size  Total: 97  Male: 55  Female: 42 | Type  Haemorrhagic  Phase  Acute | Design  Prospective study  Japan, Asia | V-VST  Baseline Test  < 24 hours | Dysphagia  (n = 57)  No dysphagia  (n = 40) | NA | NA |
| Smithard, Smeeton & Wolfe, 2007  [33] | Age  69.6  Sample size  Total: 1188  Male: 567  Female: 621 | Type  Ischaemic  Haemorrhagic  Phase  Acute | Design  Prospective study  United Kingdom, Europe | WST  Baseline Test  < 7 days | Dysphagia  (n = 567)  No dysphagia  (n = 621) | Diabetes mellitus  Hypertension  LACS  PACS  POCS  TACS | Mortality |
| Sundar et al., 2008 [34] | Age  65  Sample size  Total: 50  Male: 63  Female: 27 | Type  Ischaemic  Phase  Acute | Design  Prospective study  India, Asia | SSA  Baseline Test  < 48 hours | Dysphagia  (n = 21)  No dysphagia  (n = 29) | NA | NA |
| Suntrup et al., 2011 [35] | Age  71.1 (10.0)  Sample size  Total: 30  Male: 16  Female: 14 | Type  Haemorrhagic  Phase  Acute | Design  Prospective study  Germany, Europe | FEES  Baseline Test  < 72 hours | Dysphagia  (n = 23)  No dysphagia  (n = 7) | NA | NA |
| Suntrup et al., 2015 [36] | Age  73.7 (30-96)  Sample size  Total: 200  Male: 101  Female: 99 | Type  Ischaemic  Haemorrhagic  Phase  Acute | Design  Prospective study  Germany, Europe | FEES  Baseline Test  < 96 hours | Dysphagia  (n = 165)  No dysphagia  (n = 35) | NA | NA |
| Szu et al., 2017  [37] | Age  62.8  Sample size  Total: 130  Male: 79  Female: 51 | Type  Ischaemic  Haemorrhagic  Phase  Acute | Design  Prospective study  China, Asia | SSA  Baseline Test  < 48 hours | Dysphagia  (n = 83)  No dysphagia  (n = 47) | NA | NA |
| Teasell et al., 2001 [38] | Age  56  Sample size  Total: 20  Male: 15  Female: 5 | Type  Ischaemic  Haemorrhagic  Phase  Acute | Design  Retrospective study  Canada, North America | VFSS  Baseline Test  < 48 hours | Dysphagia  (n = 11)  No dysphagia  (n = 9) | NA | Pneumonia |
| Toscano et al., 2015 [39] | Age  73 (11.6)  Sample size  Total: 275  Male: 138  Female: 137 | Type  Ischaemic  Haemorrhagic  Phase  Acute | Design  Prospective study  Italy, Europe | WST  Baseline Test  < 48 hours | Dysphagia  (n = 121)  No dysphagia  (n = 154) | Stroke severity | NA |
| Turner-Lawrence et al., 2009 [40] | Age  62 (16.2)  Sample size  Total: 84  Male: 47  Female: 37 | Type  Ischaemic  Haemorrhagic  Phase  Acute | Design  Prospective study  USA, North America | WST  Baseline Test  < 24 hours | Dysphagia  (n = 48)  No dysphagia  (n = 36) | NA | NA |
| Zhang et al., 2012 [41] | Age  71.3  Sample size  Total: 106  Male: NA  Female: NA | Type  Ischaemic  Phase  Acute | Design  Prospective study  China, Asia | WST  Baseline Test  < 48 hours | Dysphagia  (n = 20)  No dysphagia  (n = 86) | NA | Pneumonia |
| Zhang et al., 2015 [42] | Age  65  Sample size  Total: 760  Male: 394  Female: 366 | Type  Ischaemic  Haemorrhagic  Phase  Acute | Design  Prospective study  China, Asia | WST  Baseline Test  < 48 hours | Dysphagia  (n = 482)  No dysphagia  (n = 278) | NA | NA |

FEES: Fiberoptic Endoscopic Evaluation of Swallowing, LACS (lacunar syndrome), MASA: Mann Assessment of Swallowing Ability, NA: not available, PACS (partial anterior circulation syndrome), POCS (posterior circulation syndrome), SSA: Standardized Swallowing Assessment, TACS (total anterior circulation syndrome), USA: United States of America, VFSS: Videofluoroscopic Swallowing Study, V-VST: volume-viscosity swallow test, WST: Water Swallow Test

**Supplemental Table 3:** Risk of bias assessment

| Author | External validity | | | | Internal validity | | | | | |
| --- | --- | --- | --- | --- | --- | --- | --- | --- | --- | --- |
|  | Representativeness | Sampling frame | Random selection | Non-response | Data collection | Case definition | Valid Instrument | Same mode | Numerator / denominator | Overall risk of bias |
| Abubakar 2017 | 0 | 1 | 1 | 0 | 0 | 0 | 0 | 0 | 0 | 2 – Low |
| Al-Khaled 2016 | 0 | 0 | 1 | 0 | 0 | 0 | 0 | 0 | 0 | 1 – Low |
| Barer 1989 | 0 | 0 | 1 | 0 | 0 | 0 | 0 | 0 | 0 | 1 – Low |
| Braun 2019 | 0 | 0 | 1 | 0 | 1 | 0 | 0 | 0 | 0 | 2 – Low |
| Broadley 2003 | 1 | 1 | 1 | 0 | 0 | 0 | 0 | 0 | 0 | 3 – Low |
| Carnaby 2019 | 0 | 0 | 1 | 0 | 0 | 0 | 0 | 0 | 0 | 1 – Low |
| Cong 2012 | 1 | 0 | 1 | 0 | 1 | 1 | 0 | 0 | 0 | 4 – Moderate |
| Crary 2006 | 1 | 1 | 1 | 0 | 0 | 0 | 0 | 0 | 0 | 3 – Low |
| Crary 2013 | 1 | 1 | 1 | 0 | 0 | 0 | 0 | 0 | 0 | 3 – Low |
| De Cock 2020 | 0 | 0 | 1 | 0 | 0 | 0 | 0 | 0 | 0 | 1 – Low |
| Diendere 2018 | 1 | 1 | 1 | 0 | 0 | 0 | 0 | 0 | 0 | 3 – Low |
| Ding 2019 | 1 | 1 | 1 | 0 | 1 | 0 | 1 | 0 | 0 | 5 – Moderate |
| Fenarndez-Pombo 2019 | 0 | 1 | 1 | 0 | 0 | 0 | 0 | 0 | 0 | 2 – Low |
| Gandolfo 2019 | 0 | 0 | 1 | 0 | 0 | 0 | 0 | 0 | 0 | 1 – Low |
| Gordon 1987 | 1 | 1 | 1 | 0 | 0 | 0 | 0 | 0 | 0 | 3 – Low |
| Gottlieb 1996 | 1 | 1 | 1 | 0 | 0 | 0 | 0 | 0 | 0 | 3 – Low |
| Guyomard 2009 | 1 | 1 | 1 | 0 | 0 | 0 | 0 | 0 | 0 | 3 – Low |
| Hamidon 2006 | 1 | 1 | 1 | 0 | 0 | 0 | 0 | 0 | 0 | 3 – Low |
| Hasan 2010 | 1 | 1 | 1 | 0 | 0 | 0 | 0 | 0 | 0 | 3 – Low |
| Henke 2017 | 1 | 1 | 1 | 0 | 1 | 0 | 0 | 0 | 0 | 4 – Moderate |
| Hernandez-Bello 2018 | 1 | 1 | 1 | 0 | 0 | 0 | 0 | 0 | 0 | 3 – Low |
| Khedr 2021 | 0 | 0 | 1 | 0 | 0 | 0 | 0 | 0 | 0 | 1 – Low |
| Kwon 2006 | 1 | 1 | 1 | 0 | 0 | 0 | 0 | 1 | 0 | 4 – Moderate |
| Lendinez-Mesa 2017 | 1 | 1 | 1 | 0 | 1 | 0 | 0 | 0 | 0 | 4 – Moderate |
| Li 2020 | 1 | 1 | 1 | 0 | 1 | 0 | 0 | 0 | 0 | 4 – Moderate |
| Mann 2001 | 1 | 1 | 1 | 0 | 0 | 0 | 0 | 0 | 0 | 3 – Low |
| Nam 2017 | 1 | 1 | 1 | 0 | 1 | 0 | 0 | 0 | 0 | 4 – Moderate |
| Odderson 1995 | 1 | 1 | 1 | 0 | 0 | 0 | 0 | 0 | 0 | 3 – Low |
| Paciaroni 2004 | 0 | 1 | 1 | 0 | 0 | 0 | 0 | 0 | 0 | 2 – Low |
| Rofes 2018 | 1 | 1 | 0 | 0 | 0 | 0 | 0 | 0 | 0 | 2 – Low |
| Schelp 2014 | 1 | 1 | 1 | 0 | 0 | 0 | 0 | 0 | 0 | 3 – Low |
| Shibakazi 2014 | 1 | 1 | 1 | 0 | 0 | 1 | 0 | 0 | 0 | 4 – Moderate |
| Smithard 2007 | 0 | 0 | 1 | 0 | 0 | 0 | 0 | 0 | 0 | 1 – Low |
| Sundar 2008 | 1 | 1 | 1 | 0 | 0 | 0 | 0 | 0 | 0 | 3 – Low |
| Suntrup 2011 | 1 | 1 | 1 | 0 | 0 | 0 | 0 | 0 | 0 | 3 – Low |
| Suntrup 2015 | 1 | 0 | 0 | 0 | 0 | 0 | 0 | 0 | 0 | 1 – Low |
| Szu 2017 | 0 | 0 | 1 | 0 | 0 | 0 | 0 | 1 | 0 | 2 – Low |
| Teasel 2011 | 1 | 1 | 1 | 0 | 1 | 0 | 0 | 0 | 0 | 4 – Moderate |
| Toscano 2015 | 1 | 1 | 1 | 0 | 0 | 0 | 0 | 0 | 0 | 3 – Low |
| Turner-Lawrence 2009 | 1 | 1 | 1 | 0 | 0 | 0 | 0 | 0 | 0 | 3 – Low |
| Zhang 2012 | 1 | 1 | 1 | 0 | 0 | 1 | 1 | 1 | 0 | 6 – Moderate |
| Zhang 2016 | 0 | 1 | 1 | 0 | 0 | 0 | 0 | 0 | 0 | 2 – Low |

Note: Score for each item is 0 for low risk and 1 for high risk. 0-3: low risk study, 4-6: moderate risk study, 7-9: high risk study

**Supplemetal Table 4:** Moderator analysis

| Variables | p-value | n | Prevalence (95% CI) |
| --- | --- | --- | --- |
| Age | 0.615 | 43 | -0.01 (-0.05-0.03) |
| Continent | 0.263 | 43 |  |
| South America |  | 2 | 0.61 (0.38 – 0.81) |
| North America |  | 6 | 0.46 (0.40 – 0.53) |
| Europe |  | 17 | 0.44 (0.35 – 0.54) |
| Africa |  | 4 | 0.39 (0.30 – 0.49) |
| Asia |  | 14 | 0.37 (0.27 – 0.47) |
| Stroke type | **0.0001** | 43 |  |
| Haemorrhagic |  | 3 | 0.61 (0.54 – 0.68) |
| Combined |  | 26 | 0.49 (0.42 – 0.55) |
| Ischaemic |  | 14 | 0.28 (0.23 – 0.33) |
| Assessment method | **0.0001** | 43 |  |
| Instrumental assessment |  | 4 | 0.75 (0.66 – 0.82) |
| Clinical subjective assessment |  | 6 | 0.50 (0.42 – 0.58) |
| Clinical objective assessment |  | 33 | 0.38 (0.32 – 0.44) |
| Study Quality | **0.002** | 43 |  |
| High quality |  | 33 | 0.47 (0.41 – 0.53) |
| Moderate quality |  | 10 | 0.29 (0.21 – 0.38) |
| Study design | 0.237 | 43 |  |
| Prospective |  | 30 | 0.46 (0.40 – 0.52) |
| Cross-sectional |  | 5 | 0.41 (0.24 – 0.60) |
| Retrospective |  | 8 | 0.32 (0.20 – 0.47) |

CI: confidence interval, n: number of studies.

**Supplemental Figure 1:** Funnel plot for publication bias


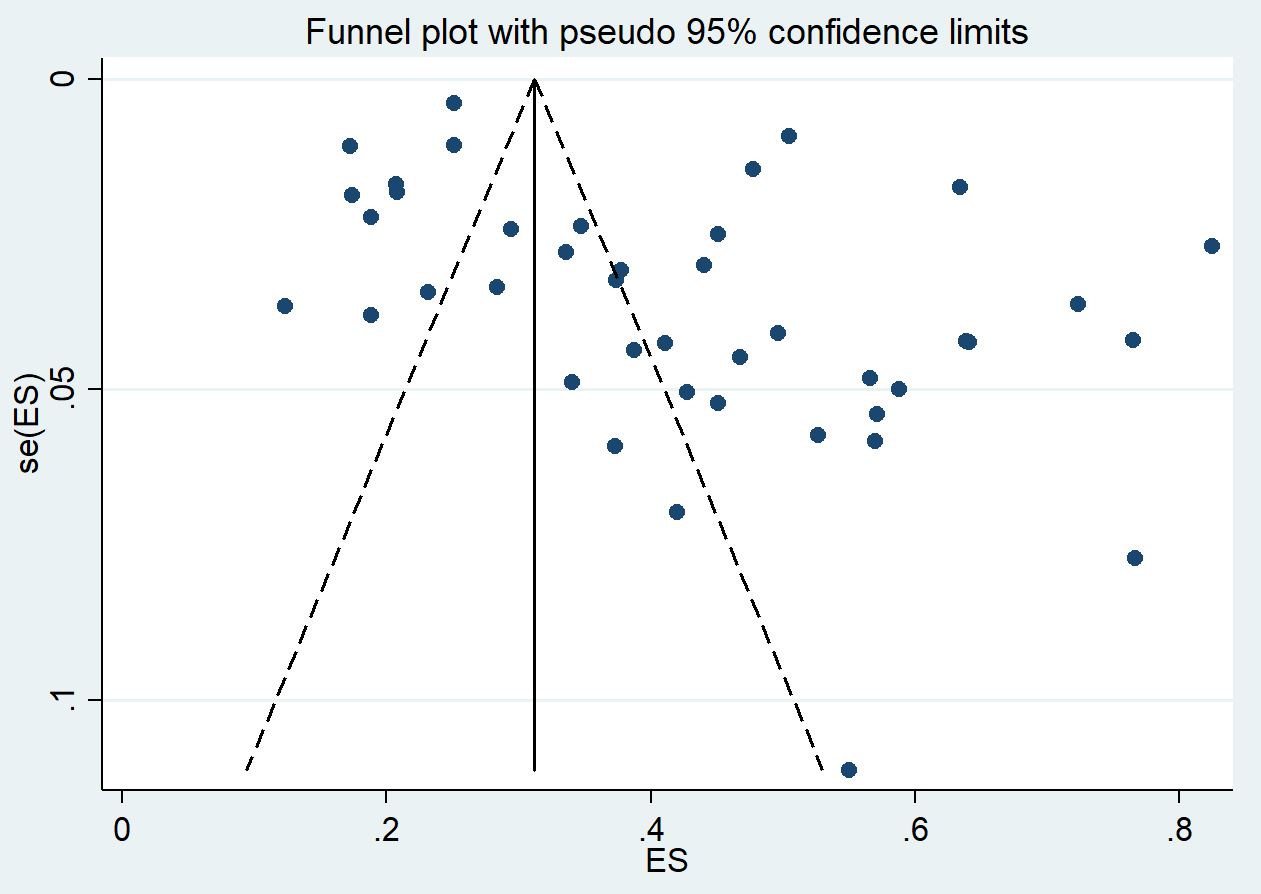


**Supplemental Figure 2:** Gender - female


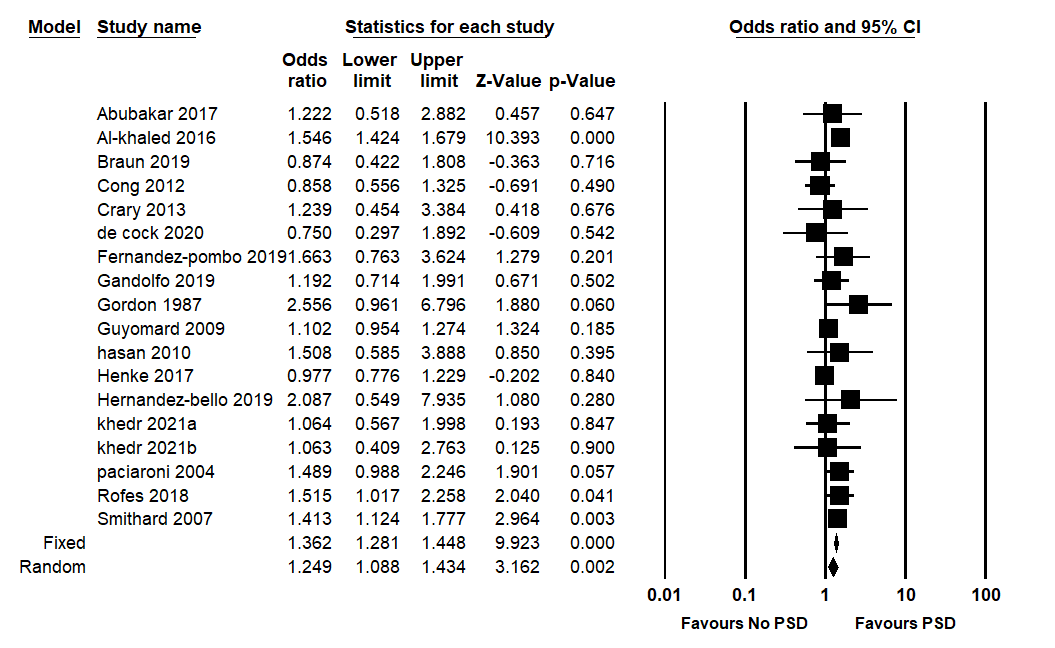


**Supplemental Figure 3:** Gender - male


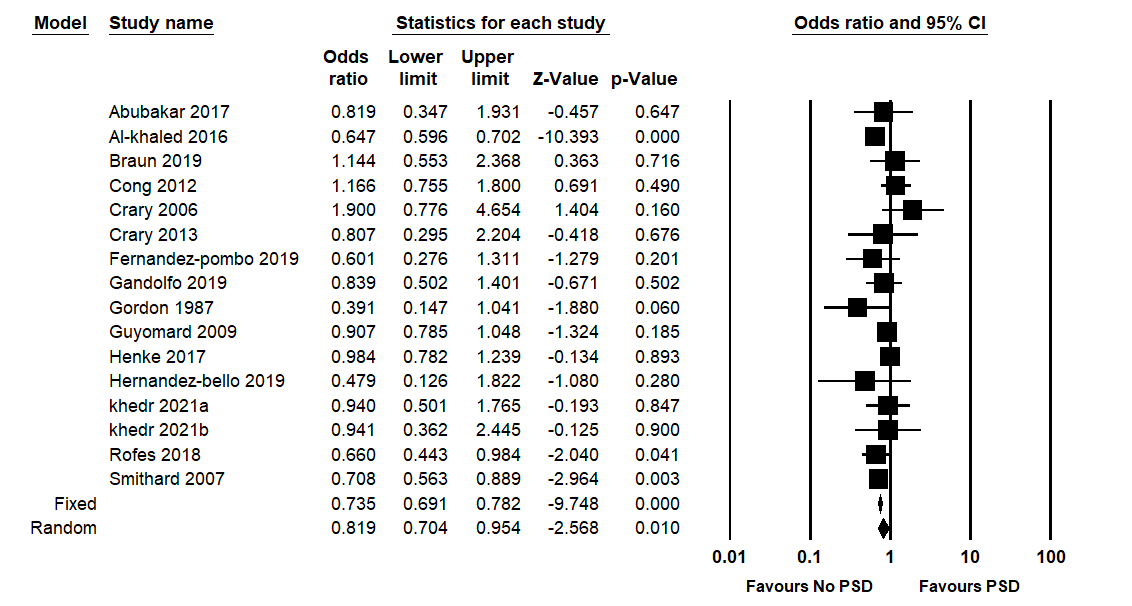


**Supplemental Figure 4:** Haemorrhagic stroke


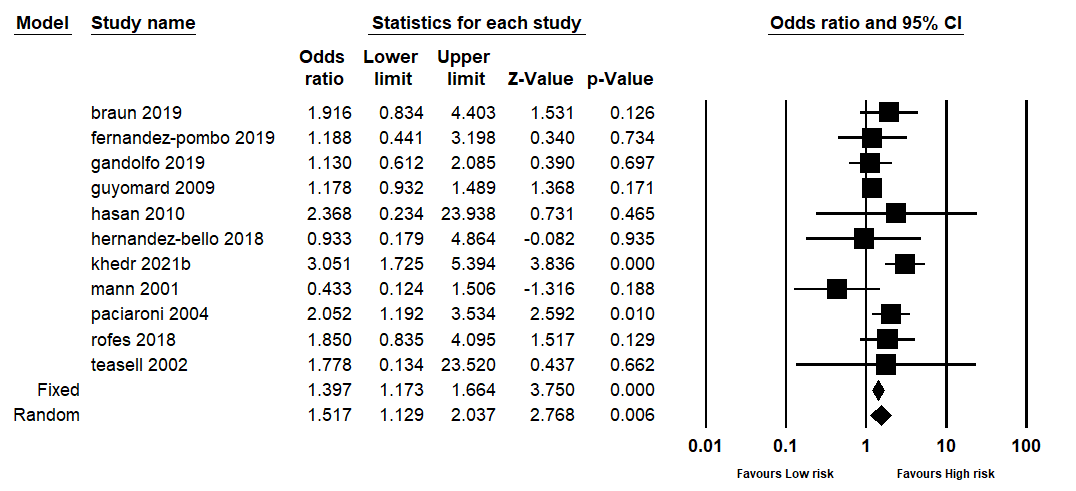


**Supplemental Figure 5:** Ischaemic stroke


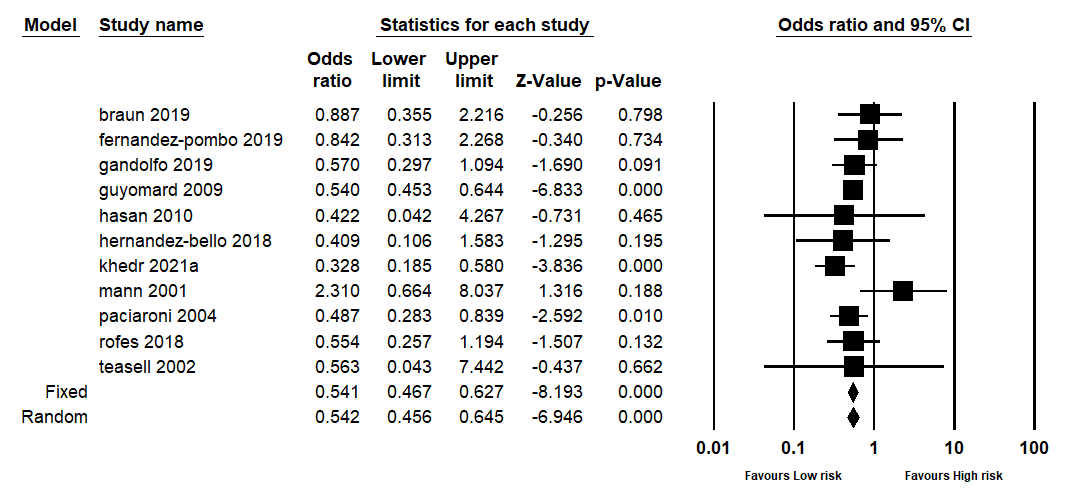


**Supplemental Figure 6:** Previous stroke


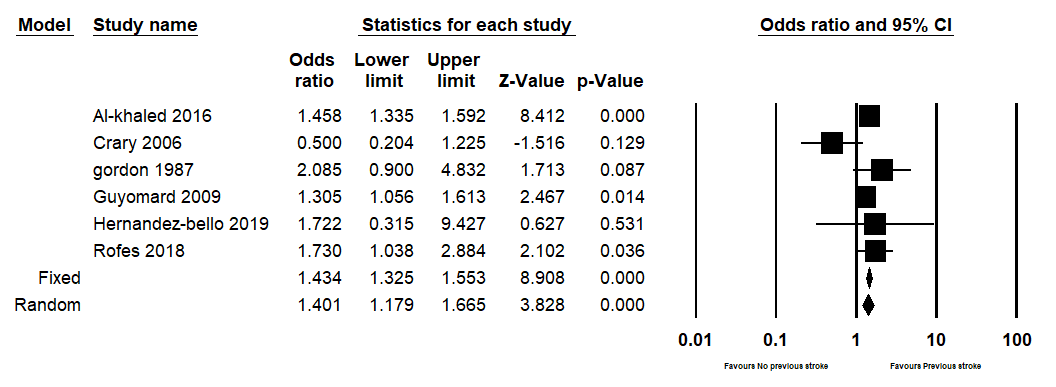


**Supplemental Figure 7:** Stroke severity


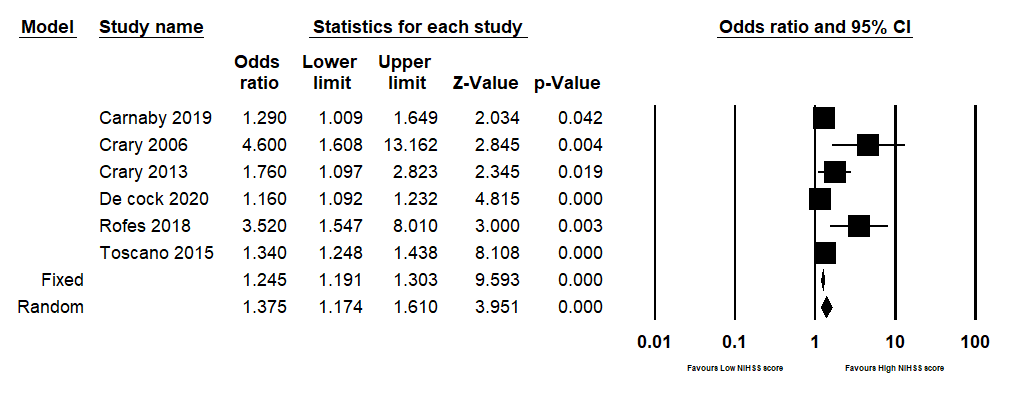


**Supplemental Figure 8:** Diabetes mellitus


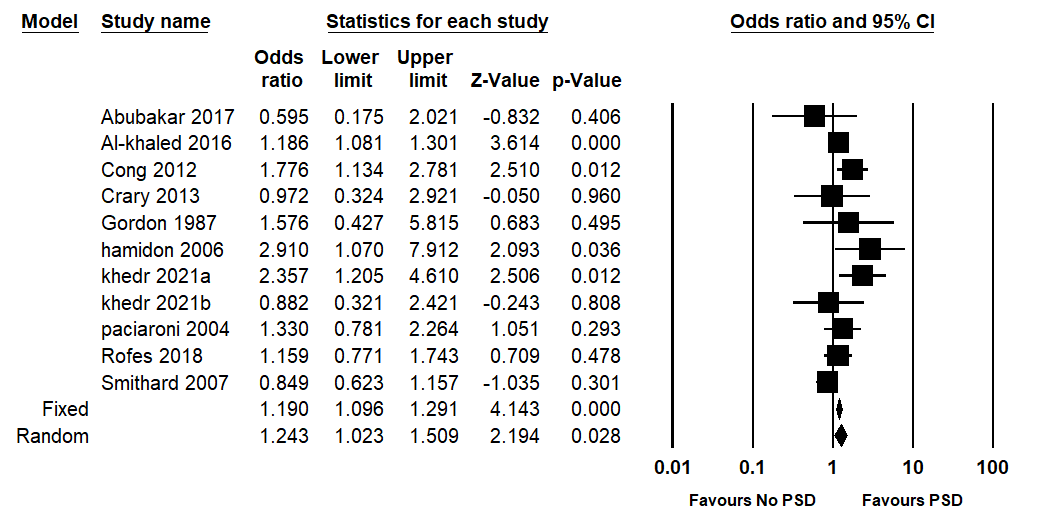


**Supplemental Figure 9:** Hypertension


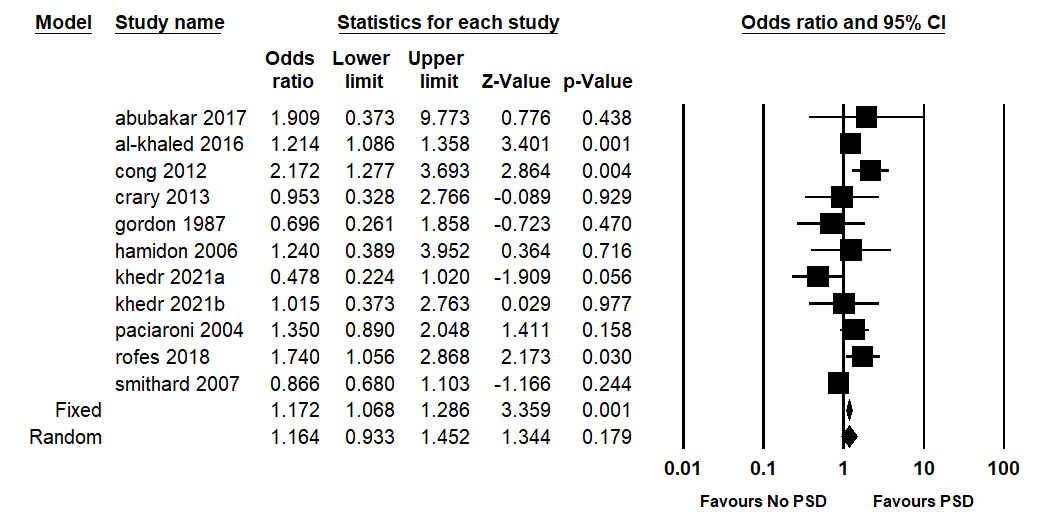


**Supplemental Figure 10:** Atrial fibrillation


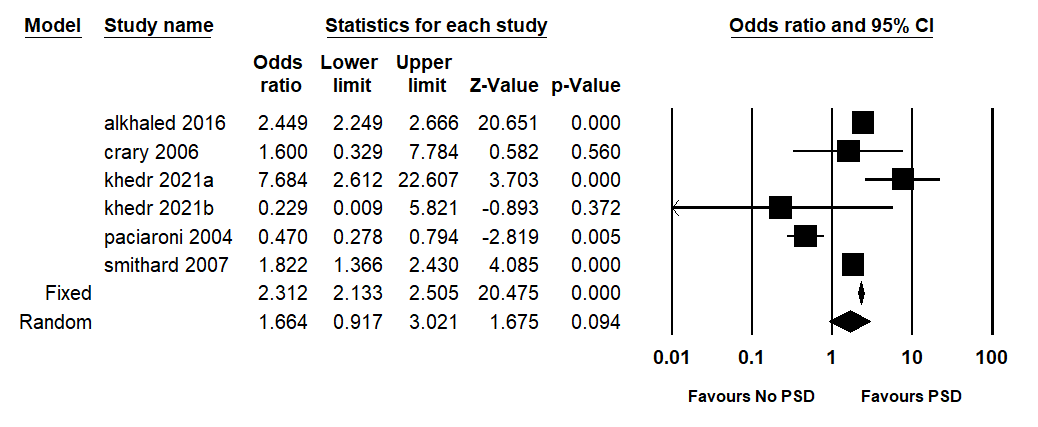


**Supplemental Figure 11:** Hyperlipidaemia


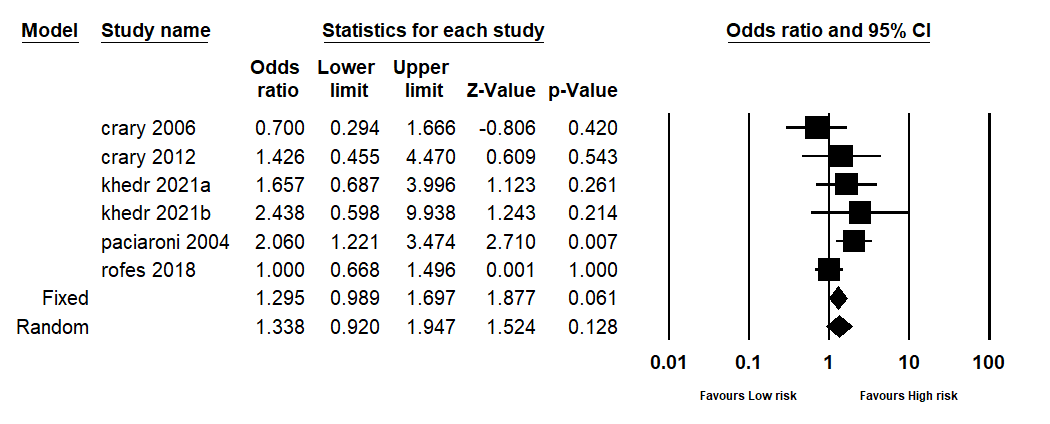


**Supplemental Figure 12:** Right hemisphere


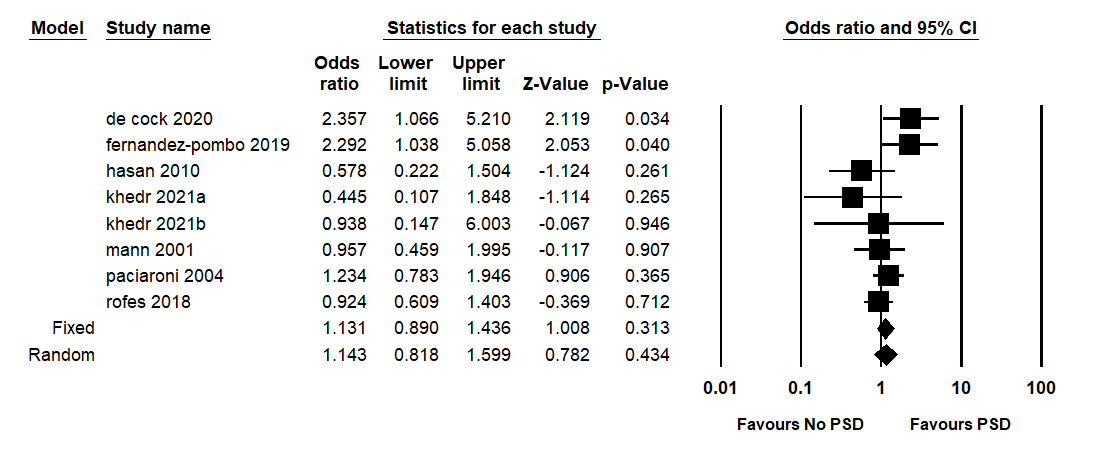


**Supplemental Figure 13:** Left hemisphere


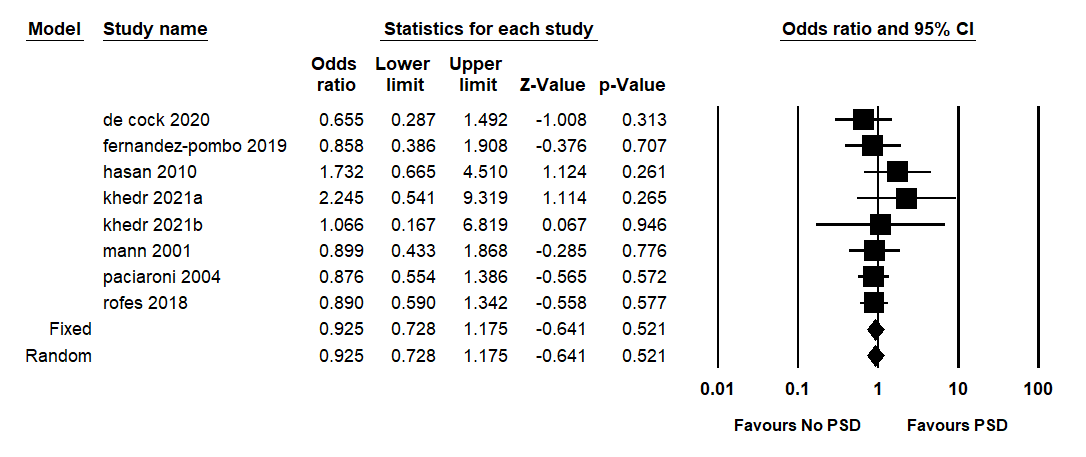


**Supplemental Figure 14:** TACS


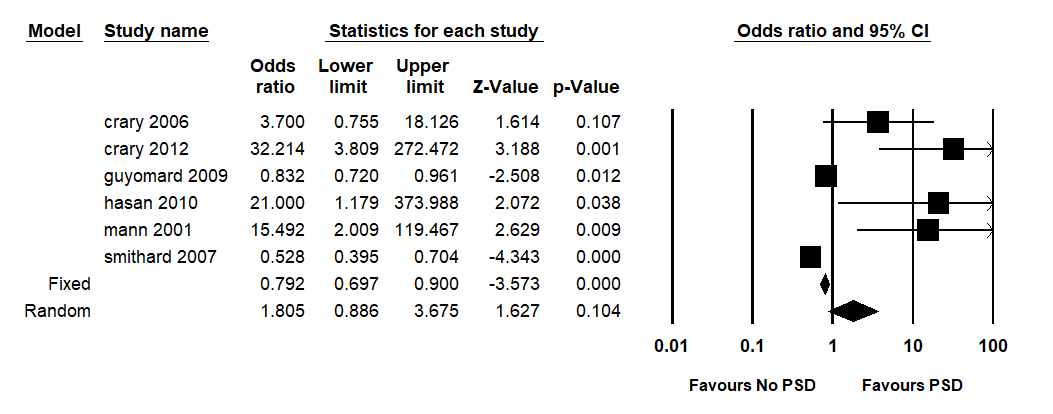


**Supplemental Figure 15:** Partial anterior circulation syndrome (PACS)


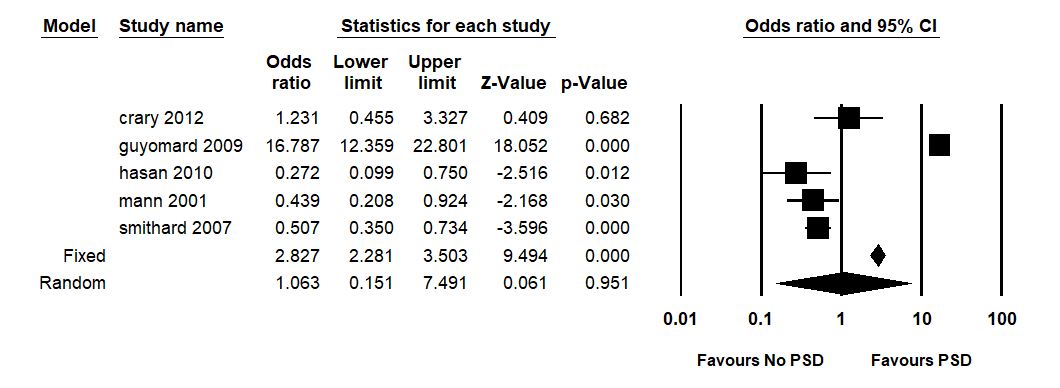


**Supplemental Figure 16:** Posterior circulation syndrome (POCS)


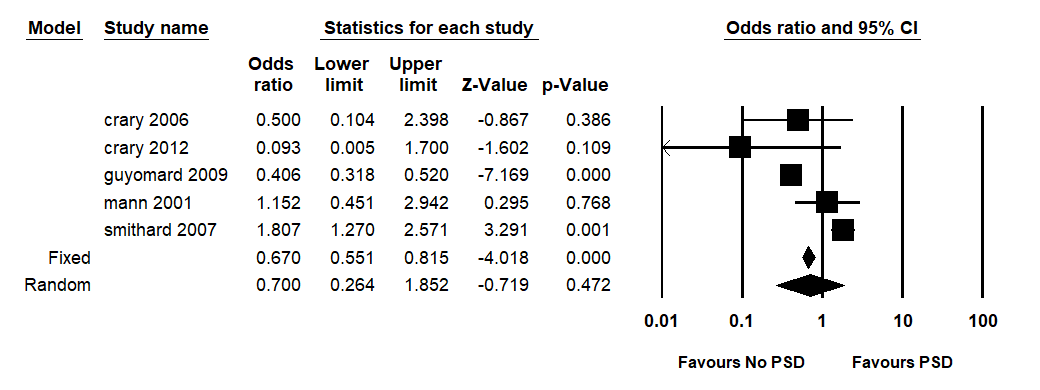


**Supplemental Figure 17:** Lacunar syndrome (LACS)


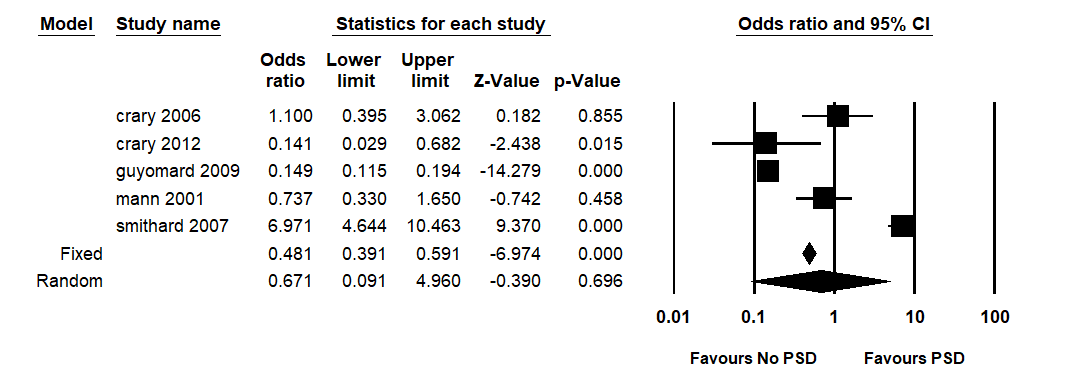


**Supplemental references**

1. Abubakar SA, Jamoh BY. Dysphagia following acute stroke and its effect on short-term outcome. Niger Postgrad Med J 2017; 24 (3): 182-186.

2. Al-Khaled M, Matthis C, Binder A et al. Dysphagia in Patients with Acute Ischemic Stroke: Early Dysphagia Screening May Reduce Stroke-Related Pneumonia and Improve Stroke Outcomes. Cerebrovasc Dis 2016; 42 (1-2): 81-89.

3. Barer DH. The natural history and functional consequences of dysphagia after hemispheric stroke. Journal of Neurology Neurosurgery and Psychiatry 1989; 52 (2): 236-241.

4. Braun T, Juenemann M, Viard M et al. Adjustment of oral diet based on flexible endoscopic evaluation of swallowing (FEES) in acute stroke patients: A cross-sectional hospital-based registry study. BMC Neurology 2019; 19 (1): 1-10.

5. Broadley S, Croser D, Cottrell M et al., Predictors of prolonged dysphagia following acute stroke. Journal of Clinical Neuroscience 2003; 10 (3): 300-305.

6. Carnaby G, Sia I, Crary M. Associations between spontaneous swallowing frequency at admission, dysphagia, and stroke-related outcomes in acute care. Archives of Physical Medicine & Rehabilitation 2019; 100 (7): 1283-1288.

7. Cong L, Jiang HT. Analysis of the risk factors associated with swallowing dysfunction in patients with ischemic stroke. Chinese Journal of Cerebrovascular Diseases 2012; 9 (8): 408-411.

8. Crary MA, Carnaby-Mann GD, Miller L, Antonios N, Silliman S. Dysphagia and nutritional status at the time of hospital admission for ischemic stroke. J Stroke Cerebrovasc Dis 2006; 15 (4): 164-71.

9. Crary MA, Humphrey JL, Carnaby-Mann G, Sambandam R, Miller L, Silliman S. Dysphagia, Nutrition, and Hydration in Ischemic Stroke Patients at Admission and Discharge from Acute Care. Dysphagia 2013; 28 (1): 69-76.

10. De Cock E, Batens K, Hemelsoet D, Boon P, Oostra K, De Herdt V. Dysphagia, dysarthria and aphasia following a first acute ischaemic stroke: incidence and associated factors. European Journal of Neurology, 2020; 27 (10): 2014-2021.

11. Diendéré J, Millogo A, Philippe F et al. Post-stroke complications and mortality in burkinabè hospitals: relationships with deglutition disorders and nutritional status. Dysphagia 2021; 36 (1):85-95.

12. Ding Y, Yan Y, Niu J et al. Braden scale for assessing pneumonia after acute ischaemic stroke. BMC Geriatrics 2019; 19 (1):1-7.

13. Fernández-Pombo A, Seijo-Raposo IM, López-Osorio N et al. Lesion location and other predictive factors of dysphagia and its complications in acute stroke. Clinical Nutrition ESPEN 2019; 1 (33): 178-182.

14. Gandolfo C, Sukkar S, Ceravolo MG et al. The predictive dysphagia score (PreDyScore) in the short- and medium-term post-stroke: a putative tool in PEG indication. Neurological Sciences 2019; 40 (8): 1619-1626.

15. Gordon C, Hewer RL, Wade DT. Dysphagia in acute stroke. British Medical Journal Clinical Research Ed 1987; 295 (6595): 411-414.

16. Gottlieb D, Kipnis M, Sister E, Vardi Y, Brill S. Validation of the 50 ml3 drinking test for evaluation of post-stroke dysphagia. Disability & Rehabilitation 1996. 18 (10): 529-532.

17. Guyomard V, Fulcher RA, Redmayne O, Metcalf AK, Potter JF, Myint PK. Effect of dysphasia and dysphagia on inpatient mortality and hospital length of stay: a database study. J Am Geriatr Soc 2009; 57 (11): 2101-2106.

18. Hamidon BB, Nabil I, Raymond AA. Risk factors and outcome of dysphagia after an acute ischaemic stroke. Med J Malaysia 2006; 61 (5): 553-557.

19. Hasan ZN, Al-Shimmery EK, Taha MA. Evaluation of neurogenic dysphagia in Iraqi patients with acute stroke. Neurosciences (Riyadh) 2010; 15 (2): 90-96.

20. Henke C, Foerch C, Lapa S. Early Screening Parameters for Dysphagia in Acute Ischemic Stroke. Cerebrovasc Dis 2017; 44 (5-6): 285-290.

21. Hernández-Bello E, Castellot-Perales L, Tomás-Aznar C. Assessment of dysphagia with the V-VST in patients hospitalised after a stroke. Revista Cientifica de la Sociedad Espanola de Enfermeria Neurologica 2019 (49): 8-15.

22. Khedr EM, Abbass MA, Soliman RK, Zaki AF, Gamea A. Post-stroke dysphagia: frequency, risk factors, and topographic representation: hospital-based study. The Egyptian Journal of Neurology, Psychiatry and Neurosurgery 2021; 57 (1): 1-8.

23. Kwon HM, Jeong SW, Lee SH, Yoon BW. The pneumonia score: a simple grading scale for prediction of pneumonia after acute stroke. American journal of infection control 2006; 34 (2): 64-68.

24. Lendinez-Mesa A, del Carmen Díaz-García M, Casero-Alcázar M, Grantham SJ, de la Torre-Montero JC, Fernandes-Ribeiro AS. Prevalence of oropharyngeal dysphagia inpatients related with cerebrovascular disease at a neurorehabilitation unit. Revista Cientifica de la Sociedad Espanola de Enfermeria Neurologica 2017; 45: 3-8.

25. Li J, Wang Y, Sun X et al. AND score: a simple tool for predicting infection in acute ischemic stroke patients without a ventilator in the Chinese population. Journal of International Medical Research 2020; 48 (3): 0300060519888303.

26. Mann G, Hankey GJ. Initial clinical and demographic predictors of swallowing impairment following acute stroke. Dysphagia 2001; 16 (3): 208-215.

27. Nam KW, Kwon HM, Lim JS, Lee YS. Leukoaraiosis is associated with pneumonia after acute ischemic stroke. BMC Neurology 2017; 17 (1): 1-5.

28. Odderson IR, Keaton JC, McKenna BS. Swallow management in patients on an acute stroke pathway: quality is cost effective. Archives of physical medicine and rehabilitation 1995; 76 (12): 1130-1133.

29. Paciaroni M, Mazzotta G, Corea F et al. Dysphagia following stroke. European Neurology 2004; 51 (3): 162-167.

30. Rofes L, Muriana D, Palomeras E et al. Prevalence, risk factors and complications of oropharyngeal dysphagia in stroke patients: a cohort study. Neurogastroenterology & Motility 2018; 30 (8): e13338.

31. Schelp AO, Cola PC, Gatto AR, Silva RG, Carvalho LR. Incidence of oropharyngeal dysphagia associated with stroke in a regional hospital in São Paulo State - Brazil. Arquivos de Neuro-Psiquiatria 2004; 62 (2 B): 503-506.

32. Shibazaki K, Kimura K, Aoki J, Uemura J, Fujii S, Sakai K. Dysarthria plus dysphagia is associated with severe sleep-disordered breathing in patients with acute intracerebral hemorrhage. European Journal of Neurology 2014; 21 (2): 344-348.

33. Smithard DG, Smeeton NC, Wolfe CD. Long-term outcome after stroke: does dysphagia matter? Age Ageing 2007; 36 (1): 90-94.

34. Sundar U, Pahuja V, Dwivedi N, Yeolekar ME. Dysphagia in acute stroke: Correlation with stroke subtype, vascular territory and in-hospital respiratory morbidity and mortality. Neurology India 2008; 56 (4): 463-470.

35. Suntrup S, Warnecke T, Kemmling A et al. Dysphagia in patients with acute striatocapsular hemorrhage. Journal of Neurology 2012; 259 (1): 93-99.

36. Suntrup S, Kemmling A, Warnecke T et al. The impact of lesion location on dysphagia incidence, pattern and complications in acute stroke. Part 1: Dysphagia incidence, severity and aspiration. European Journal of Neurology 2015; 22 (5): 832-838.

37. Szu LY, Hsieh SI, Tseng SM, Huang TH. The determinants of dysphagia in patients with stroke during hospitalized rehabilitation. Hu Li Za Zhi The Journal of Nursing 2017; 64 (3): 43-55.

38. Teasell R, Foley N, Fisher J, Finestone H. The incidence, management, and complications of dysphagia in patients with medullary strokes admitted to a rehabilitation unit. Dysphagia 2002; 17 (2): 115-120.

39. Toscano M, Cecconi E, Capiluppi E et al. Neuroanatomical, clinical and cognitive correlates of post-stroke dysphagia. European Neurology 2015; 74 (3-4): 171-177.

40. Turner-Lawrence DE, Peebles M, Price MF, Singh SJ, Asimos AW. A feasibility study of the sensitivity of emergency physician Dysphagia screening in acute stroke patients. Annals of Emergency Medicine 2009; 54 (i3): 344-348.

41. Zhang X, Wang F, Zhang Y, Ge Z. Risk factors for developing pneumonia in patients with diabetes mellitus following acute ischaemic stroke. J Int Med Res 2012; 40 (5): 1860-1865.

42. Zhang J, Zhao X, Wang A et al. Emerging malnutrition during hospitalisation independently predicts poor 3-month outcomes after acute stroke: data from a Chinese cohort. Asia Pacific Journal of Clinical Nutrition 2015; 24 (3): 379-386.
